# Identification and analysis of MKK and MPK gene families in canola (*Brassica napus* L.)

**DOI:** 10.1186/1471-2164-14-392

**Published:** 2013-06-11

**Authors:** Wanwan Liang, Bo Yang, Bao-Jun Yu, Zili Zhou, Cui Li, Ming Jia, Yun Sun, Yue Zhang, Feifei Wu, Hanfeng Zhang, Boya Wang, Michael K Deyholos, Yuan-Qing Jiang

**Affiliations:** 1 State Key Laboratory of Crop Stress Biology for Arid Areas and College of Life Sciences, Northwest A & F University, Yangling, Shaanxi 712100, China; 2 Department of Biological Sciences, University of Alberta, Edmonton T6G 2E9, Canada

**Keywords:** Abiotic stress, Biotic stress, *Brassica napus*, MKK, MPK, *Sclerotinia sclerotiorum*, WRKY

## Abstract

**Background:**

Eukaryotic mitogen-activated protein kinase (MAPK/MPK) signaling cascades transduce and amplify environmental signals via three types of reversibly phosphorylated kinases to activate defense gene expression. Canola (oilseed rape, *Brassica napus*) is a major crop in temperate regions. Identification and characterization of MAPK and MAPK kinases (MAPKK/MKK) of canola will help to elucidate their role in responses to abiotic and biotic stresses.

**Results:**

We describe the identification and analysis of seven *MKK* (*BnaMKK*) and 12 *MPK* (*BnaMPK*) members from canola. Sequence alignments and phylogenetic analyses of the predicted amino acid sequences of BnaMKKs and BnaMPKs classified them into four different groups. We also examined the subcellular localization of four and two members of BnaMKK and BnaMPK gene families, respectively, using green fluorescent protein (GFP) and, found GFP signals in both nuclei and cytoplasm. Furthermore, we identified several interesting interaction pairs through yeast two-hybrid (Y2H) analysis of interactions between BnaMKKs and BnaMPKs, as well as BnaMPK and BnaWRKYs. We defined contiguous signaling modules including BnaMKK9-BnaMPK1/2-BnaWRKY53, BnaMKK2/4/5-BnaMPK3/6-BnaWRKY20/26 and BnaMKK9-BnaMPK5/9/19/20. Of these, several interactions had not been previously described in any species. Selected interactions were validated *in vivo* by a bimolecular fluorescence complementation (BiFC) assay. Transcriptional responses of a subset of canola MKK and MPK genes to stimuli including fungal pathogens, hormones and abiotic stress treatments were analyzed through real-time RT-PCR and we identified a few of *BnaMKKs* and *BnaMPKs* responding to salicylic acid (SA), oxalic acid (OA), *Sclerotinia sclerotiorum* or other stress conditions. Comparisons of expression patterns of putative orthologs in canola and Arabidopsis showed that transcript expression patterns were generally conserved, with some differences suggestive of sub-functionalization.

**Conclusions:**

We identified seven *MKK* and 12 *MPK* genes from canola and examined their phylogenetic relationships, transcript expression patterns, subcellular localization, and protein-protein interactions. Not all expression patterns and interactions were conserved between canola and Arabidopsis, highlighting the limitations of drawing inferences about crops from model species. The data presented here provide the first systematic description of MKK-MPK-WRKY signaling modules in canola and will further improve our understanding of defense responses in general and provide a basis for future crop improvement.

## Background

Environmental stresses including salinity, extreme temperatures, limited water availability, and fungal pathogens deeply limit agricultural productivity. Alleviating these problems and ensuring sustainable agricultural development is a priority for plant biologists. During their evolution, plants have acquired a complex defense system to responsd to adverse environmental conditions. The mitogen-activated protein kinase (MAPK/MPK) cascades, which are composed of three groups of protein kinases: MAPKK kinases (MAPKKKs, MAP3Ks, or MEKKs), MAPK kinase (MAPKKs, MAP2Ks, MKK or MEKs) and MAPK/MPKs, are conserved in eukaryotes through evolution and operate as sequential signal transducers via phosphorylation to amplify, channel and integrate information from the cellular environment to the transcriptional response and metabolic response centers [1,2].

In Arabidopsis, there are 80 MAPKKK, 10 MKK and 20 MPK genes [3,4]. The ten MKK genes in Arabidopsis may activate different MPKs and hence may integrate cross-talk of different signaling pathways. MKKs can be classified into four major groups (A, B, C and D) based on their S/TxxxxxS/T consensus domain and “D sites” [4]. AtMKK1, -2, -4 and -5 have been reported to play roles in plant stress responses [5-8]. The MPK families also have four major groups (A, B, C and D) based on their conserved T-D/E -Y motif with groups A, B, C harboring T-E-Y while group D has T-D-Y motif [4]. Arabidopsis MPK3, -4 and -6, which are in group A and B respectively, are involved in many diverse processes including developmental processes and stress responses [9-12].

WRKY transcription factors (TFs) are important transcriptional regulators that modulate immune responses and abiotic stresses [13]. Emerging evidence demonstrates that group I WRKY transcription factors, which contain a conserved SP cluster and/or D domain motif in their N-terminal regions, could be activated by MAPK-dependent phosphorylation, suggesting post-translational regulation of WRKY transcription factors [14]. So far, several MAPKKK-MKK-MPK and MPK-WRKY modules and their functions have been well-studied. For instance, Arabidopsis MPK4 is phosphorylated by upstream MKK1/2 and MEKK1 and, once phosphorylated, it phosphorylates and activates MAP kinase 4 substrate 1 (MKS1), which then triggers the dissociation of the inactivated form of MPK4-MKS1-WRKY33 to release MKS1-WRKY33 [12,15-18] The released MKS1-WRKY33 can then enter the nucleus to initiate the transcription of downstream *PHYTOALEXIN DEFICIENT3* (*PAD3*), required for the antimicrobial camalexin production [12,18]. On the other hand, AtMPK3 and -6 are activated by upstream MKK4 and -5 to phosphorylate WRKY33 and, through transcriptional regulation of the camalexin biosynthetic genes PAD2 and PAD3, confer resistance to fungal pathogens [19]. A more recent report provided direct evidence that AtWRKY33 is phosphorylated by AtMPK3 and AtMPK6 *in vivo* in responses to *Botrytis cinerea* infection [20], suggesting that phosphorylation of WRKY transcription factors by MPKs could be an important means to transduce the signal to nuclei.

Expression of a constitutively active AtMPK4 (CA-MPK4) reduced SA accumulation and defense resistance against bacteria *Pseudomonas syringae* pv tomato strain DC3000 (*Pst DC3000*) [21]. Moreover, pathogen-associated molecular pattern (PAMP)-triggered reactive oxygen species (ROS) were also repressed in CA-MPK4 plants, which is different from the effects regulated by either MPK4 or the loss-of-function *mpk4* mutant [21]. Arabidopsis MEK1-MPK6 was also involved in abscisic acid (ABA) and sugar signaling in the process of seed germination, as the induction of *NCED3* and *ABA2* by sugar was abolished in the double mutant *mek1/mpk6*[22]. AtMPK3/6-AtWRKY33 regulates 1-amino-cyclopropane-1-carboxylic acid synthase (ACS) activity, a rate-limiting step in ethylene biosynthesis pathway, at both transcriptional and protein stability levels in plant immune responses to *B. cinerea*[23]. AtMPK3 and -6 are also involved in stomatal dynamics and development [24]. Stomatal closure caused by drought and other stresses is mediated by the phytohormone ABA and H_2_O_2_ and, this process also requires the participation of the AtMKK1 and AtMPK3 and -6 [25]. AtMKK3 could act upstream of group C MPKs (MPK1, -2, -7 and -14) to regulate the downstream pathogenesis-related (PR) genes since *ProPR1*::*GUS* expression was enhanced by the co-expression of *AtMKK3/AtMPK7*. *AtMKK3*-overexpression lines with increased PR gene expression are tolerant while the knock-out *mkk3* lines are susceptible to *Pst DC3000*[26].

MAPK signaling cascades have also been reported to play important roles in other plant species. A MAPK from rice (*Oryza sativa*), BWMK1, can phosphorylate a transcription factor, OsEREBP1, and overexpressing *BWMK1* in tobacco enhanced expression of many pathogenesis-related genes with enhanced resistance to pathogens [27]. *OsMAPK5* is induced by various biotic (pathogen infection) and abiotic (wounding, drought, salt, and cold) stresses; however, overexpression or RNAi-mediated suppression demonstrated that OsMAPK5 can positively regulate drought, salt, and cold tolerance while negatively modulating PR gene expression and resistance to fungal (*Magnaporthe grisea*) and bacterial (*Burkholderia glumae*) pathogens [28]. A more recent report shows that OsMKK6 phosphorylates OsMPK3 and these constitute a moderately (12°C) but not a severely (4°C) low temperature signalling pathway [29]. In *Nicotiana benthamiana* and *Nicotiana tabacum*, SA-induced protein kinase (SIPK) and wound-induced protein kinase (WIPK) are well-known to be involved into osmotic stress, wounding, and biotic stress [30,31]. In *Zea mays*, ZmMPK3 and -5 are reported to participate in the plant response to cold, drought, ultraviolet light and oxidative stress [32,33]. In *Triticum aestivum*, *TaMPK3* and *-6* are differentially regulated at multiple levels during compatible disease interactions with *Mycosphaerella graminicola*[34]. In *Solanum lycopersicum* (*Lycopersicon peruvianum*), LeMPK1 and LeMPK2 were activated in response to systemin, four different oligosaccharide elicitors (OEs), and UV-B radiation [35]. In *Medicago sativa*, SIMK, MMK2, MMK3, and SAMK were activated by excess copper or cadmium ions. Furthermore, SIMK is also activated by SIMK kinase (SIMKK). However, there are still many unknown MAPKKK-MKK-MPK modules waiting to be identified and characterized [36]. So far, no systematic investigations of MAPKK/MKK and MAPK/MPK gene families have been reported in *Brassica napus*.

Canola (oilseed rape, *Brassica napus* L.) is a major oil crop in temperate regions and its quality and quantity are often limited by adverse environmental stresses including fungal pathogens. The fungal disease sclerotinia stem rot, caused by *S. sclerotiorum* (Lib.) de Bary, is a particular problem in canola [37]. Due to the lack of resistant germplasm, and the fact that the resistance is possibly mediated by multiple genes, it is necessary to identify and explore the genes that contribute to plant resistance against *S. sclerotiorum*[38-42]. In our previous transcriptional study of canola infected with *S. sclerotiorum*, we identified several WRKY genes and *MPK3, -4, -6* and *-17* as well as several MKK and MAPKKK genes that are responsive to *S. sclerotiorum* infection [43]. Further, from canola, we identified 46 and cloned 38 WRKY genes and studied them [44]. However the biological significance and functions of most of the canola MKK and MPK genes, except *MPK4*, have not been described so far in *B. napus*[11]. In the present study, we describe the identification and cloning of seven BnaMKK and 12 BnaMPK genes and, analyze them using bioinformatics and molecular and biochemical assays. We found that some of the BnaMKK and BnaMPK genes are responsive to multiple stress treatments including *S. sclerotiorum*, oxalic acids (OA), drought, etc. Through yeast two-hybrid (Y2H) assay, both BnaMKK-BnaMPK and BnaMPK-BnaWRKY interactions were screened and several interesting MAPK modules were identified. Finally, comparison of expression patterns of putative orthologs from canola and Arabidopsis showed that they may be involved in different signalling pathways. Through this work, we can better understand the biotic (defense) and abiotic stress responses as well as the molecular mechanisms regualted by MKK-MPK-WRKY modules in canola. The data presented here would also lay a solid foundation for improving the tolerance of canola against *S. sclerotiorum* and other abiotic stresses through modulating the expression level of these genes.

## Results and discussion

### Identification, cloning of *BnaMKK* and *BnaMPK* genes in canola

As the first step to understand the roles of MKK and MPK signaling cascades in canola response to abiotic and biotic stresses, we aimed to clone *BnaMKK* and *BnaMPK* genes from canola. Since the sequencing of *Brassica napus* genome is not finished and Arabidopsis is a close relative to *B. napus*, we used the sequences of 10 MKK genes and 20 MPK genes of Arabidopsis as queries, and ran BLAST searches of the expressed sequence tag (EST) database (release 110101) of *B. napus* in NCBI. As a result, we identified 373 ESTs representing MPKs and 58 ESTs for MKKs (Table 1, Additional file 1: Table S1), which showed significant similarities with an e-value lower than 10^-4^. These ESTs were manually curated and then assembled to obtain contigs and singlets, which were then reciprocally BLAST searched against the Arabidopsis transcript database, TAIR10 (http://www.arabidopsis.org/Blast/index.jsp) to identify the putative orthologs. The names of canola MKK and MPK genes were assigned based on names of their presumptive Arabidopsis orthologs. Afterwards, the amino acids of each contig or singlet were predicted using DNAMAN or DNASTAR program. As a result, we successfully identified ESTs representing 8 *BnaMKK* and 18 *BnaMPK* genes (Table 1, Additional file 1: Table S1). To facilatate the characterization of these two gene families, we named each gene with a three-letter code starting with *Bna* (for *B. napus*) followed the family designation (MKK or MPK), and finally a number consistent with the Arabidopsis MAPK and MAPKK nomenclature (Table 1).

**Table 1:** ***BnaMPKs *and *BnaMKKs *identified in this study**

**Gene**	**GenBank Acc No.**^**a**^	**EST count**	**AA No.**	**pI value**	**Arabidopsis ortholog**^**b **^**/AGI No.**	**Rice ortholog**^**b**^**/locus**	**Subcellular localization**^**c**^			
							**PSORT**	**CELLO**	**ESLPred**	**experimental**
*BnaMKK1*	JQ708028	3	356	6.75	AtMKK1/ At4g26070	OsMKK1/Os06g05520.1	nuc	nuc	cyt	
*BnaMKK2*	JQ708029	3	365	5.99	AtMKK2/ At4g29810	N/A	cyt	cyt, nuc	cyt	cyt, nuc
*BnaMKK3*	JQ708030	3	519	5.58	AtMKK3/ At5g40440	OsMKK3/Os06g27890.1	cyt, nuc	cyt, pm	cyt	cyt, nuc
*BnaMKK4*	JQ708031	4	357	9.2	AtMKK4 /At1g51660	OsMKK5/Os06g09180.1	nuc, cyt	nuc	nuc	cyt, nuc
*BnaMKK5*	KC246595	2	333	8.89	AtMKK5/ At3g21220	OsMKK4/Os02g54600.1	mit	nuc	nuc	
*BnaMKK6*	JQ708032	2	357	6.19	AtMKK6/ At5g56580	OsMKK6/Os01g32660.1	cyt	nuc, cyt	cyt	
*BnaMKK8*	No	1			AtMKK7/ At5g56581					
*BnaMKK9*	JQ708033	1	307	8.22	AtMKK9/ At1g73500	N/A	chl, nuc	mit	cyt	
*BnaMPK1*	JQ708034	2	371	6	AtMPK1/ At1g10210	OsMPK7/Os06g48590.1	nuc	cyt	cyt	
*BnaMPK2*	JQ708038	5	374	6.6	AtMPK2/ At1g59580	N/A			cyt	
*BnaMPK3*	JQ708040	4	371	6	AtMPK3/ At3g45640	OsMPK3/Os03g17700.1	nuc, cyt	cyt	nuc	cyt, nuc
*BnaMPK4*	JQ708041	7	374	6.06	AtMPK4/ At4g01370	OsMPK4/Os10g38950.1	cyt	cyt	nuc	
*BnaMPK5*	JQ708042	3	374	5.7	AtMPK5/ At4g11330	OsMPK2/Os08g06060.1	cyt	cyt	nuc	cyt, nuc
*BnaMPK6*	JQ708043	6	396	5.19	AtMPK6/ At2g43790	OsMPK6/Os06g06090.1	nuc	cyt	nuc	cyt, nuc
*BnaMPK7*	No	13			AtMPK7/ At2g18170					
*BnaMPK8*	JQ708044	12	582	6.65	AtMPK8/ At1g18150	N/A	nuc	cyt	nuc	
*BnaMPK9*	JQ708045	1	502	8.42	AtMPK9/ At3g18040	OsMPK17-1/Os06g49430.1	cyt, nuc	cyt	cyt	cyt, nuc
*BnaMPK10*	No	2			AtMPK10/At3g59790					
*BnaMPK12*	No	4			AtMPK12/At2g46070					
*BnaMPK13*	No	1			AtMPK13/At1g07880					
*BnaMPK15*	No	1			AtMPK15/At1g73670					
*BnaMPK16*	JQ708035	12	559	8.8	AtMPK16/At5g19010	OsMPK/Os05g05160.1	cyt	cyt	cyt	
*BnaMPK17*	JQ708036	10	488	6.83	AtMPK17/At2g01450	N/A	cyt	cyt	cyt	
*BnaMPK18*	No	32			AtMPK18/At1g53510					
*BnaMPK19*	JQ708037	7	600	9.2	AtMPK19/At3g14720	N/A	cyt	pp,cyt	cyt	
*BnaMPK20*	JQ708039	10	612	9.23	AtMPK20/At2g42880	OsMPK20-1/Os01g43910.1	cyt	cyt	mit	

^a^The full-length cDNA was cloned and deposited in GenBank. No, cloning not sucessful.

^b^Putative orthologs were identified by InParanoid (http://inparanoid.sbc.su.se/cgi-bin/index.cgi) with a maximum score. N/A, homolog but not ortholog was identified.

^c^Subcellular localizations are predicted using three different programs, one is PSORT (http://psort.nibb.ac.jp), the second is CELLO v2.5 (http://cello.life.nctu.edu.tw) and the third is ESLPred (http://www.imtech.res.in/raghava/eslpred/index.html). nuc-nuclear, cyt-cytoplasm,pp-perplasm, mit-mitochondria,chl-chloroplast,and pm-plasma membrane.

We used RT-PCR and RACE (rapid amplification of cDNA ends) to clone the cDNA sequences of the *BnaMKK* and *BnaMPK* genes using high-fidelity polymerase. As a result, seven *BnaMKK* and 12 *BnaMPK* genes were cloned and the sequences were deposited in the GenBank (Table 1). Orthologs from different species may have similar biological functions [45]. Because functions of some MKK and MPK genes in the model plants Arabidopsis and rice have been well studied, we compared our cloned *BnaMKK* and *BnaMPK* sequences to those of Arabidopsis and rice and identified presumed orthologs in each species (Table 1).

### Phylogenetic analysis, multiple alignment and domain analysis of BnaMKKs and BnaMPKs

To better understand the evolutionary history of both MKK and MPK gene families, we also retrieved MKK and MPK gene sequences from a variety of species representing major land plant lineages including the bryophyte *Physcomitrella patens* (Pp), the lycophyte *Selaginella moellendorffii* (Sm), and several mono- and eudicotyledonous angiosperms, namely: the eudicots *Arabidopsis thaliana* (At), *Vitis vinifera* (Vv), *Populus trichocarpa* (Pt) and *Glycine max* (Gm); and the monocots *Oryza sativa* (Os), *Sorghum bicolor* (Sb), *Brachypodium distachyon* (Bd), and *Zea mays* (Zm). By searching the green alga *Chlamydomonas reinhartdii* (Cr) genome in Phytozome v9.0, we also identified two MKK genes, whose protein sequences showed great similarity to AtMKK3 and AtMKK6, respectively (Additional file 2: Table S2). Furthermore, we also identified an MKK gene from a marine green alga *Ostreococcus tauri* (*Ot*), which is the world’s smallest free-living eukaryote known to date [46]. It should be noted that based on evidence from ESTs, the size of the canola MKK protein family is roughly comparable to that of either Arabidopsis, rice or poplar, although not all MKK genes have been cloned from canola. On the other hand, the green alga *C. reinhartdii* and lower land plants *P. patens* and *S. moellendorffii* apparently evolved a smaller set of MKKs relative to the flowering plants, which indicates an expansion of MKK gene family after the divergence of flowering plants from the other lineages.

**Figure 1:**
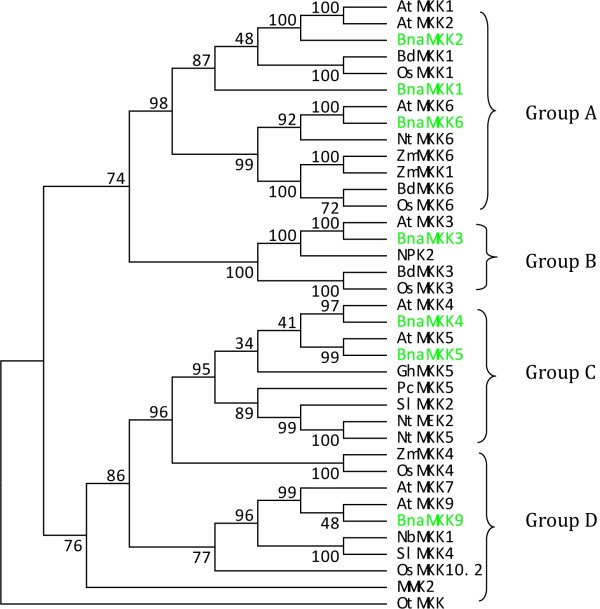
**Phylogenetic analysis of MKKs and domain analysis of seven BnaMKKs.** Phylogenetic relationship of BnaMKKs with MKKs from other species. The respective MKK proteins are depicted by a two to three-letter code denoting the species in combination with numbers indicating the specific MKK gene from each species. The phylogenetic tree was reconstructed using the maximum parsimony method of MEGA5.1 program. The green highlighted proteins are from canola. The analysis involved 39 amino acid sequences. There are a total of 594 positions in the final dataset. OtMKK, an MKK from the marine green alga *Ostreococcus tauri* (*Ot*) was used to root the tree. The numbers on the nodes are percentages from a bootstrap analysis of 500 replicates. At, *A. thaliana;* Bd*, Brachypodium distachyon;* Bna, *Brassica napus*; Cr, *Chlamydomonas reinhartdii*; *Gh, G. hirsutum; Nt, N. tabacum; Ot, O. tauri; Os, O. sativa; Pc,P. crispum; Sl, S. lycopersicum* and *Zm, Z. mays.*

**Figure 2:**
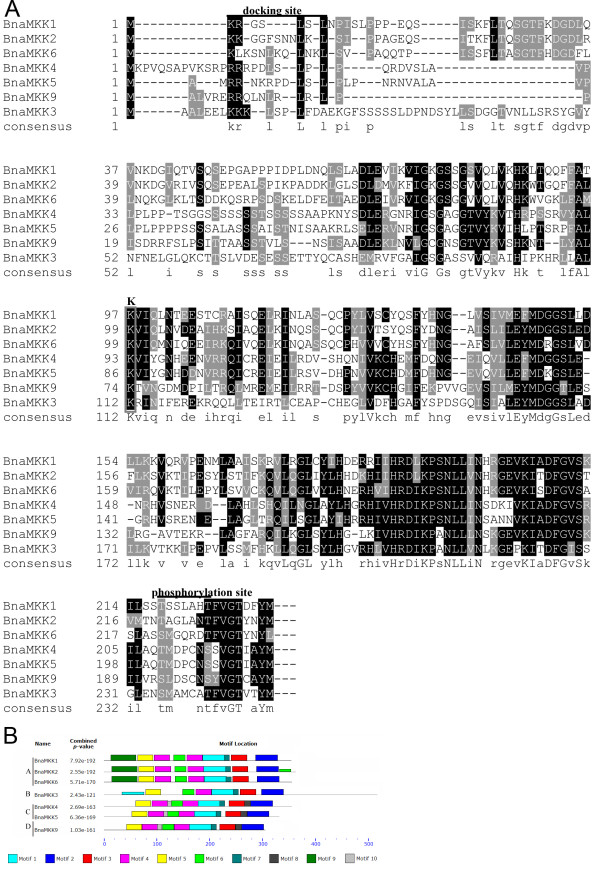
**Sequence analysis of BnaMKKs. (A)** Partial amino acid alignment of the seven BnaMKK proteins. Multiple sequence alignment was performed using the ClustalX1.83 and illustrated by BOXSHADE (http://www.ch.embnet.org/software/BOX_form.html). Identical amino acids are shaded in black, and similar amino acids are shaded in gray. The docking and phosphorylation sites are indicated by overbars, with the lysine (K) necessary for the kinase activity is boxed. **(B)** Schematic diagram of amino acid motifs of BnaMKKs. Motif analysis was determined by using MEME4.0 program as described in Methods. Different colors of the boxes represent 10 motifs in the corresponding position of each BnaMKK proteins. The information of 10 motifs was illustrated in Additional file 4: Figure S2.

The amino acid sequences of MKKs collected from 21 species, together with our BnaMKK sequences, were used to construct a phylogenetic tree using the maximum parsimony (MP) method (Figure 1, Additional file 3: Figure S1). The OtMKK was used to root the tree. As shown by the tree’s topology, the MKK proteins from various species could be divided into four major groups, each supported by highly significant bootstrap values. The seven canola MKKs were distributed in each of the four groups, with BnaMKK1, -2 and -6 belonging to Group A, BnaMKK3 Group B, BnaMKK4 and -5 Group C, and BnaMKK9 Group D (Figure 1), which is consistent with previous phylogenetic analysis of MKKs from Arabidopsis, rice and poplar [1]. Furthermore, the seven BnaMKK members were always clustered in the same subgroup closely with AtMKK orthologs, which was expected for genes from these two representatives of the Brassicaceae family. The multiple alignment of the collected BnaMKKs showed that they each have a putative MPK docking sites (D sites) at the N terminus, characterized by a cluster of basic residues (R or K) N-terminal to hydrophobic residues (L or I) (Figure 2A), as reported for the MKKs from model species [4]. These BnaMKK proteins also contain a highly conserved phosphorylation site, which has a consensus sequence S/TxxxxxS/T, as previously identified in Arabidopsis MKK proteins (Figure 2A).

The conserved motifs among the BnaMKKs were further analyzed using the MEME4 program, and a schematic of the motifs is presented (Figure 2B, Additional file 4: Figure S2a and b). Seven motifs were conserved in all BnaMKKs identified, while motifs 7, 8, 9 and 10 were group-specific. Group A (BnaMKK1, -2 and -6) has motif 9 in the N-terminal sequence especially, while only group C (BnaMKK4 and -5) and D (BnaMKK9) had motif 10. BnaMKK2 had an extra motif 6 in the C terminal region. In addition, we found that BnaMKK3 had a long C-terminal region and a similar observation was also made with BdMKK3 [47]. The motifs from MEME analysis were further annotated by comparisons with known protein kinase motifs/domains (http://prosite.expasy.org/scanprosite/). We noted that motif 1 contained both IiHrDLKpsNLLV, which is the active-site signature of serine/threonine protein kinases, and S/TXXXXS/T, which is the consensus sequence [4]. Motif 5 contains protein kinases ATP-binding signature, IGKGSSGVVQlVqhkwtgqf, in which a glycine-rich loop (G-x-G-x-x-G) is required for ATP binding. Motif 7 (VGTxxYMSPER) is the conserved signature in the catalytic domains as described previously [4].

**Figure 3:**
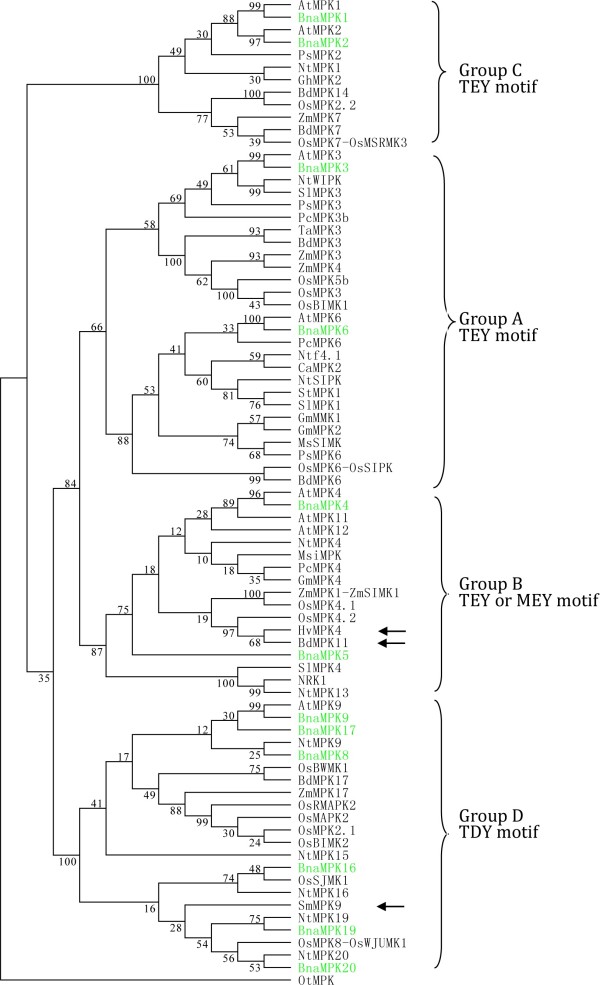
**Phylogenetic analysis of MPKs and domain analysis of 12 BnaMPKs.** Phylogenetic relationship of BnaMPKs with MKKs from other species. The respective MPK proteins are depicted by a two to three-letter code denoting the species in combination with numbers representing the exact MKK from this species. The phylogenetic tree was reconstructed through maximum parsimony method of MEGA5.1 program. The green highlighted proteins are BnaMPKs. The analysis involved 85 amino acid sequences. There are a total of 752 positions in the final dataset. The numbers on the nodes are percentages from a bootstrap analysis of 500 replicates. The atypical MEY motif in the activation loop of a few MPKs is marked by arrows. OtMPK is used as the outgroup. At, *A. thaliana;* Bd*, Brachypodium distachyon;* Bna, *Brassica napus*; Ca*, C. annuum;* Gm*, G. max;* Gh*,G. hirsutum;* Ms*, M. sativa;* Msi*, Malus sieversii;* Nt,*N. tabacum;* Os*,O. sativa;* Ot*,O. tauri;* Pc*,P. crispum;* Ps, *P. sativum;* Pt*, P. trichocarpa*; Sl, *S. lycopersicum;* Ta*,T. aestivum and* Zm*, Z. mays.*

To further explore the evolutionary history of the MAPK family in plants, we retrieved MPK sequences from a various plant species (Additional file 2: Table S2). We identified a single MPK gene from *O. tauri* (*Ot*) and used its protein sequence to root a bootstrapped consensus tree. We performed a phylogenetic analysis of the 12 BnaMPKs in the context of MPK proteins retrieved from a representative selection of species (Figure 3) and then in all the MPK sequences collected (Additional file 5: Figure S3). We found that the identified 12 BnaMPKs could be assigned unambiguously to four separate clades, together with 20 AtMPKs [4]. Group A included BnaMPK3 and -6, group B includes BnaMPK4 and -5 and, group C includes BnaMPK1 and -2, whereas six BnaMPKs belong to D, which are BnaMPK8, -9, -16, -17, -19 and -20. Most MPKs are classified into group D instead of group A, B or C, which is partially in agreement with previous study in rice [48]. This indicates that gene loss may have occurred in groups A to C, after the monocot-dicot divergence [48]. As expected, most of the BnaMPKs were clustered more closely with Arabidopsis than those of other species.

**Figure 4:**
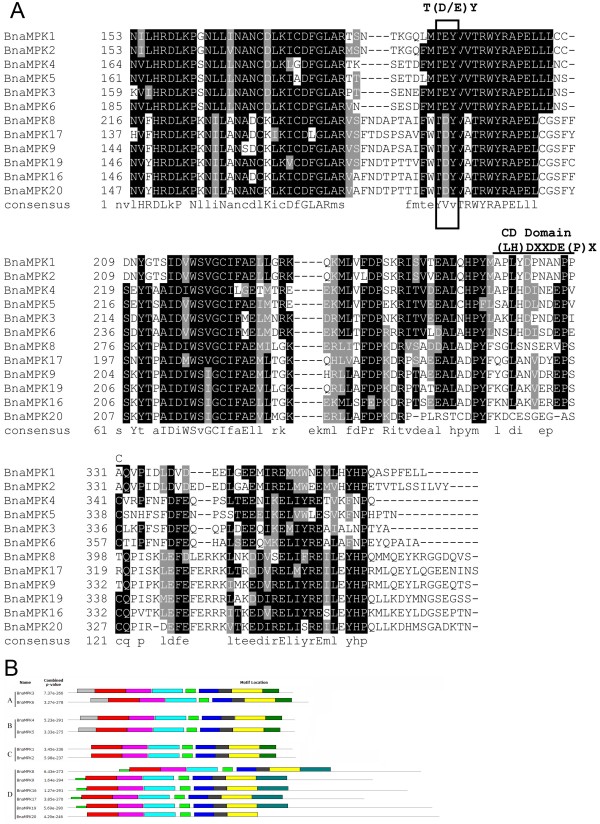
**Sequence analysis of BnaMPKs. (A)** Partial amino acid alignment of the 12 BnaMPK members. Multiple sequence alignment was performed using the ClustalX1.83 and illustrated by BOXSHADE (http://www.ch.embnet.org/software/BOX_form.html). Identical amino acids are shaded in black, and similar amino acids are shaded in gray. The phosphorylation-activation motif (TDY and TEY) was highlighted by the box and the PK sub-domains of VII and VIII were labelled as above. **(B)** Schematic diagram of amino acid motifs of BnaMPKs. Motif analysis was determined by using MEME4.0 program as described in Methods. Different colors of the boxes represent 10 motifs in the corresponding position of each BnaMKK proteins. The information of 10 motifs was illustrated in Additional file 7: Figure S5.

MPKs are located at the terminus of prototypical sequential cascades, and can be phosphorylated and therefore activated by MKKs via dual phosphorylation of conserved threonine and tyrosine residues in the motif TxY, which is located in the activation loop (T-loop) between subdomains VII and VIII of MPKs [4]. Each of the 12 BnaMPKs we identified possessed a TEY or TDY signature (Figure 4A). A MEY motif was identified in BdMPK11 [47] and also MPK from other species including SmMPK9, HvMPK4 and NtMPK4. Since *S. moellendorffii* is a member of an ancient vascular plant lineage that first appeared in the fossil record some 400 million years ago [49], the presence of an atypical MEY-type activation loop in this species suggests its early divergence. As can be seen from Figure 4A and Additional file 5: Figure S3, the TEY-type MPKs can be classified into three groups, A, B and C, whereas all the TDY-type MPKs form a more distinct group D, as described previously [4]. Interestingly, in the ancient unicellular green alga *O. tauri*, we also identified a single MPK, which contains a TEY motif, suggesting that the common ancestor of eukaryotic MPKs may have been of TEY type. During the long history of evolution and selective forces, MPK proteins may have acquired other types of TxY motif besides the canonical TEY motif [50]. In addition, the aforementioned three atypical MEY-type MPK proteins we identified fell into the TEY-B clade in the tree, whereas SmMPK9 was clustered together with TDY clade (Figure 4A, Additional file 5: Figure S3). Since all analyzed land plant lineages in the phylogenetic tree contained both TEY- and TDY-type MPKs, this indicated that an early gene duplication event likely generated the split between the two major canonical classes of TEY- and TDY-type MPKs [51].

MPKs may contain a CD domain, defined as [LH][LHY]Dxx[DE]xx[DE]EpxC, where x means any amino acid and the two adjacent acidic residues (D and E) are critical for interacting with a cluster of basic amino acids (K and R) in MKKs [52]. We found that the groups A, B and C of BnaMPKs possessed a CD or modified CD domain, while neither domain appeared in the group D (Figure 4B), which is also consistent with previous findings from other species [52]. From the alignment of amino acid sequences of OsMPKs, AtMPKs and BnaMPKs, we identified the characteristic motif of the MPK family, which is defined as Fx7Rx2Rex8Hx28Mx3Lx20QxLx6HxAx3HRDLKPxNx6C and is conserved among rice, Arabidopsis and canola as well as *O. tauri*, except for AtMPK1 and its ortholog BnaMPK1 (Additional file 6: Figure S4). BnaMPK1 and AtMPK1 had a Tyr (Y) instead of Phe (F) at the very beginning of the conserved motif. However, the OsMPK20.2 downloaded from the rice genome annotation project lacked this region due to an unknown reason.

The conserved motifs in the 12 BnaMPK proteins were also analyzed using MEME4, and a schematic of the motifs is presented (Figure 4B, Additional file 7: Figure S5). Seven motifs were conserved in all of the members of BnaMPK, while motifs 7, 9 and 10 are found only in some groups. For instance, Group A, B and C did not have motif 7 at the C-terminal region while group C and D did not have motif 10 at the N-terminal end. Similarly, we annotated the motifs from MEME analysis and identified that eight of the ten motifs (1, 2, 3, 4, 5, 6, 8 and 10) are actually subdomains (I-XI) of kinase domain of MPKs [51]. Motif 1 contains VI and VII subdomains, motif 2 is subdomain IX, motif 3 contains subdomain I harboring a glycine-rich loop (G-x-G-x-x-G) required for ATP binding. Motif 3 also contains subdomains II and III, which represent the active-site signature IlHrDLKpgNLLI of serine/threonine protein kinases. Motif 4 contains subdomains IV and V, motif 5 has subdomain XI and, motif 6 has subdmain VIII, which has the conserved consensus -T(D/E)Y- motif in the catalytic domain of plant kinases. Motif 8 has subdomain X. Motifs 7 and 9 contain CD domain, which is (LH)DXXDE(P)X acting as a docking site for MAPKKs [4]. Consistent with our finding in Figure 4A, the motif analysis through MEME4.0 showed that Group D did not have motif 9, which is CD domain, while Groups A, B and C had it (Figure 4B, Additional file 7: Figure S5).

From our phylogenetic analysis, multiple alignment and domain analysis of BnaMKK and BnaMPK in canola, we concluded that MKK and some of the MPK family members may be conserved among monocots while others were lost after the divergence of the monocots and dicots. In all, The MPK signaling cascades were relatively conserved during long history of evolution, and genes belonging to the same clade or group might fulfill similar functions. The phylogenetic analysis together with the domain motif analysis presented will facilitate the functional annotation and study of uncharacterized MKKs and MPKs.

### Subcellular localization of BnaMKK and BnaMPK proteins *in vivo*

**Figure 5:**
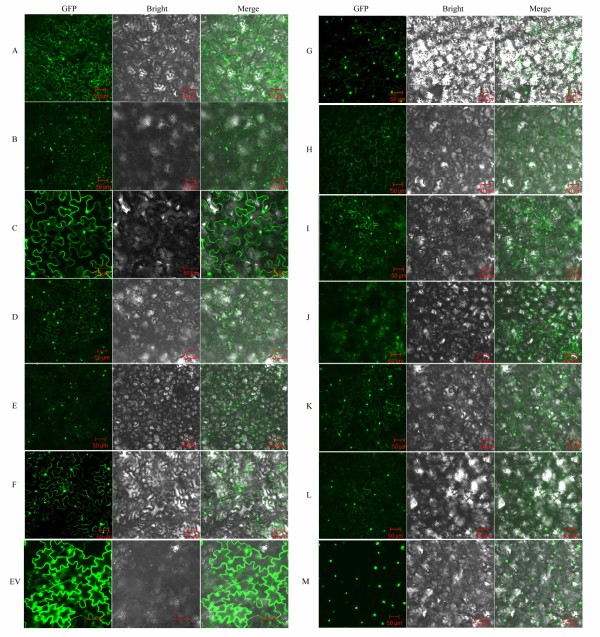
**Subcellular localization of three BnaMKK, four BnaMAPK proteins and BnaWRKY20 in *N. benthamiana *cells using green fluorescence protein (GFP).** Panels **A-E** represent BnaMKK2-sGFP **(A** and **B)** BnaMKK3-sGFP **(C)**, BnaMKK4-sGFP **(D** and **E)** and Panels **F-M** represent the subcellular localization of BnaMPK3-sGFP **(F** and **G)**, BnaMPK5-sGFP **(H)**, BnaMPK6-sGFP **(I** and **J)**, BnaMPK9-sGFP **(K** and **L)** and BnaWRKY20 **(M)** respectively. Panels B, E, G, J, and L are subcellular localization of BnaMKK2, -4, and BnaMPK3, -6, -9 under mannitol treatment (500 mM for 1 h). In each panel, the extreme left is GFP fluorescence, the middle bright field and the right an overlay of the two images. Bar = 50 μm.

Previous reports of subcellular localization of MKKs and MPKs suggest specific functions of these proteins in response to diverse stimuli [53,54]. Subcellular localization analysis of individual MAPK modules *in vivo* is therefore helpful in understanding how a single MAPK component can simultaneously mediate several responses and how a single stimulus could direct several different MAPK modules to separate tasks [54]. We first used three different programs, WoLF PSORT, CELLO v2.5 and ESLPred, to predict the subcellular localization of the 7 BnaMKKs and 12 BnaMPKs (Table 1). At the same time, we used TMHMM (http://www.cbs.dtu.dk/services/TMHMM-2.0/) to predict transmembrane helices (TMHs) of these proteins, but failed to identify any TMH (data not shown). Secondly, to examine the subcellular localization of BnaMKKs and BnaMPKs *in planta*, we randomly selected three BnaMKK genes and four BnaMKK genes for green fluorescent protein (GFP) fusion. To this end, the coding region of *BnaMKK2,-3,*and *-4* as well as *BnaMPK3, -5, -6, -9* were fused to the N-terminus of green fluorescent protein (GFP) reporter gene with a Gly-Ala rich peptide linker between CDSs and GFP in a binary vector. Then, *A. tumefaciens* cells transformed with each of these constructs and p19 protein of tomato bushy stunt virus were infiltrated into leaves of *Nicotiana benthamiana*. Two days later, under confocal microscope, we observed that both BnaMPK-GFP and BnaMKK-GFP fusion proteins emitted green fluorescence signals in nuclei and in cytoplasm of epidermal cells of leaves (Figure 5A, C, D, F, H, I and K), suggesting that the MPK and MKK proteins were localized to the cytoplasm and are small enough to diffuse or be trafficked to the nucleus. When the leaf discs were further treated with a hyperosmotic solution (500 mM mannitol) for 1 h and the plasmolyzed tissues were examined with the same settings in confocal microscope, the GFP signals were still evident in both the cytoplasm and nuclei (Figure 5B, E, G, J and L). Taken together, these results indicated that both BnaMKKs and BnaMPKs studied are localized both in the cytoplasm and nuclei. Comparing the predicted localization patterns (Table 1) to our observations using GFP (Figure 5), the results from *in silico* prediction and the *in vivo* experimental determination were generally similar, with some exception that highlight the need to examine localization *in planta*.

Recent reports have provided direct evidence that specific Arabidopsis and tobacco MPKs can phosphorylate some WRKY TFs to initiate transcription of target genes [20,55]. TFs are usually localized in the nuclei, as demonstrated by many studies including ours [44]. Therefore, we also checked the subcellular localization of a WRKY TF, BnaWRKY20, in *N. benthamiana* and the result showed that it is localized in nuclei only (Figure 5M), which is consistent with its role in gene transcription. As a control, in the *N. benthamiana* leaf cells transformed with the empty vector bearing GFP gene only, the GFP signals were also observed in both cytosol and nuclei (Figure 5EV). Therefore, the co-localization of BnaMPKs and BnaWRKYs might facilitate their interaction, phosphorylation and activation.

### Identification and validation of BnaMKK-BnaMPK and BnaMPK-BnaWRKY interactions

**Figure 6:**
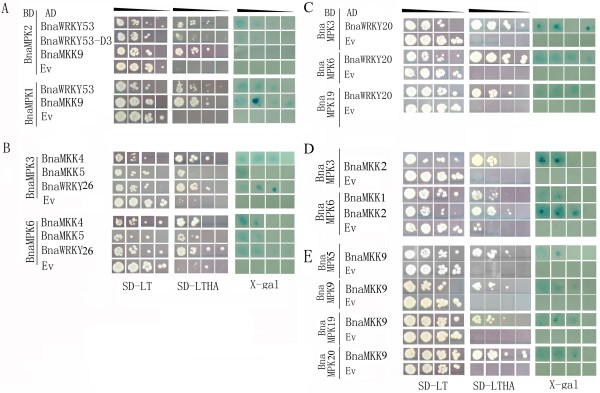
**Yeast two-hybrid (Y2H) screening of BnaMPK interaction partners.** The yeast strain AH109 harboring the indicated plasmid combinations was grown on either the nonselective (SD-LT) or selective (SD-LTHA) media, followed by X-Gal staining. Control tests were BnaMPK-pGBKT7 with empty pGADT7 vectors. Decreasing cell densities in the dilution series are illustrated by narrowing triangles. The protein interaction pairs are **(A)** BnaMPK1/2 with BnaWRKY53 and BnaMPK1/2 with BnaMKK9; **(B)** BnaMPK3/6 with BnaWRKY26 and BnaMKK4/5; **(C)** BnaWRKY20 with BnaMPK3/6/9; **(D)** BnaMPK3 with BnaMKK2, BnaMPK3/6 with BnaMKK1; **(E)** BnaMPK5/9/19/20 with BnaMKK9.

**Figure 7:**
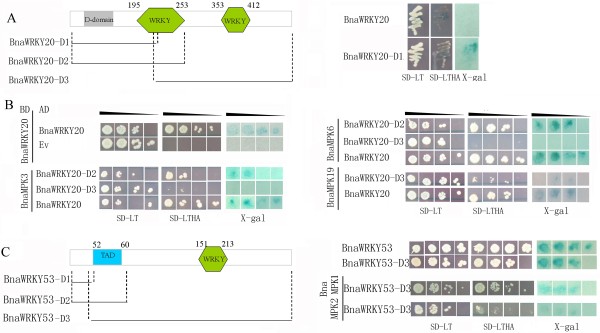
**The transactivation assay of BnaWRKY20 and -53 and their interaction domains with BnaMPKs.** Three tests (lanes 1–3) used to determine the interactors of BnaMPKs were cell grown on the nonselective (SD-LT [lane 1] or selective (SD-LTHA [lane 2]) followed by β-galactosidase assay (lane 3). Control tests were BnaMPK-pGBKT7 with empty pGADT7 vectors. **(A)** Transactivation domain analysis of BnaWRKY20; **(B)** The interaction assay of BnaWRKY20 as well as it truncated versions with BnaMPK3/6/19. For BnaWRKY20, the peptide with amino acids (AA) 29-77 is the D-domain. D1, AA1-237; D2, AA1-255; D3, AA231-532; **(C)** Transactivation assay of BnaWRKY53 as well as interaction of its full-length and truncated versions with BnaMPK1/2. For BnaWRKY53, D1, AA1-51; D2, AA1-68; D3, AA44-316; TAD, AA52-60.

So far, the best characterized MKK genes are from Arabidopsis, which has 10 MKK genes, and which activate different MPKs [4]. It is necessary to understand interactions between MKKs and MPKs to elucidate cross-talk between different signaling pathways. Therefore, to characterize components of the BnaMPK cascade as well as their upstream and downstream interaction partners, we used the yeast two-hybrid (Y2H) system to analyze interactions between seven BnaMKKs and 12 BnaMPKs, as well as interactions between BnaMPKs and BnaWRKY TFs. To do this, seven *BnaMKKs* and nine *BnaWRKYs* (*BnaWRKY1, -6, -20, -26, -28, -31, -45, -53 and -69*) were individually cloned in-frame into the pGADT7 vector while the 12 *BnaMPK* genes were cloned into pGBKT7 vector. This strategy was chosen to avoid possible transactivation of expression of marker genes (e.g. histidine synthease gene *HIS3* and β-galactosidase gene *LacZ*) by the TFs to be tested in Y2H. Only yeast colonies expressing both of the interacting MKKs and MPKs (or MPKs and WRKYs) could grow on the selection media SD-LTHA (synthetic dropout lacking leucine, tryptophan, histidine and adenine hemisulfate). Only MKK-MPK and MPK-WRKY pairs that showed interactions were reported in Figures 6 and 7. The other combinations that did not show any interaction in our Y2H assay were not presented here. The authenticity and strength of the interactions were further examined by titration assays and X-gal staining.

Our Y2H assay revealed that BnaMKK9 significantly interacted with BnaMPK1, -2, -5, -9, -19 and -20, and BnaMKK9 did not show any interaction with the other seven BnaMPKs tested. On the other hand, BnaMKK4 and -5 showed interactions with BnaMPK3 and -6 (Figure 6). When these interactions were compared to those between Arabidopsis orthologs, AtMKK9 was reported to interact with AtMPK10, 17 and -20 in Y2H [56]. Interaction between AtMKK9 and AtMPK5 was previously identified by protein microarray [57], although not validated *in planta* yet. These differences between our observations with canola proteins and previous observations using Arabidopsis genes may be attributed to either technical or biological factors. Different vectors and yeast strains were used in the canola and Arabidopsis experiments. The proteins may have also evolved to have different interactions in these two species.

As for the downstream WRKY transcription factors that BnaMPK may phosphorylate and activate, our Y2H assay showed (as also evidenced from the titration assay and X-gal assay) that BnaMPK1 and -2 significantly interacted with BnaWRKY53 and BnaMKK9 (Figure 6A). This is in agreement with a previous study showing interaction between AtMPK2 and AtWRKY53 through protein microarray profiling [57], although the interaction needs to be validated *in planta*. A previous study demonstrated that AtMEKK1 could directly regulate AtWRKY53 in the process of leaf senescence [58]. Based on the protein microarray analysis, validated in *N. benthamiana*, AtWRKY53 was phosphorylated when co-expressed with the AtMKK7/AtMPK7 module [57]. Whether this is true also for the presumed canola orthologs is yet to be confirmed, since no cDNA or EST has been identified for *BnaMKK7*. Nevertheless, our study indicates that both BnaMPK1 and -2 could act upstream of BnaWRKY53 and may therefore phosphorylate and activate BnaWRKY53 to transcribe downstream target genes in canola.

**Figure 8:**
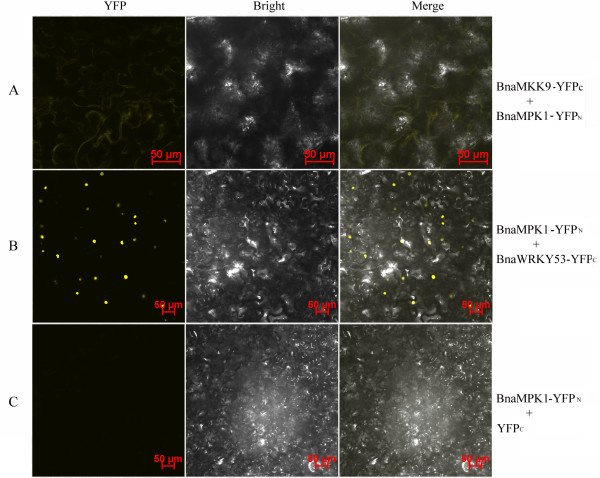
***In vivo *BiFC analysis of BnaMPK1 interacting proteins BnaMKK9 and BnaWRKY53 in co-transformed in *N. benthamiana *leaf epidermal cells.** BnaMPK1 was fused to N- terminal while **(A)** BnaMKK9 and **(B)** BnaWRKY53 were fused to C-terminal halves of YFP, individually. **(C)** A vector control with no protein fusion to the C-terminal YFP is also shown. The fluorescence of YFP was observed by confocal laser microscopy. Bar = 50 μm.

To confirm the interaction of BnaMKK9 and BnaMPK1, -2 as well as the interaction between BnaMPK1, -2 and BnaWRKY53, we performed *in vivo* bimolecular fluorescence complementation (BiFC) analysis. Yellow fluorescence signals appeared in infiltrated cells when BnaMKK9 with BnaMPK1 were co-expressed and YFP signals also appeared in nuclei when BnaMPK1 and BnaWRKY53 were co-infiltrated (Figure 8A, B). As a control, BnaMPK1 with empty vector did not show any YFP signal (Figure 8C). As we identified that BnaMKK9 interacted with BnaMPK1, -2, -5, -9, 19 and -20 (Figure 6A, E), and only BnaMPK1/2 and -19 were found to interact with BnaWRKY53 and BnaWRKY20, respectively (Figure 6A, C), the downstream targets of BnaMPK5, -9, or -20 are still waiting to be identified through Y2H screening of cDNA libraries. As BnaMPK19 was identified to interact with both upstream BanMKK9 and downstream BnaWRKY20 (Figure 6C, E), these three components BnaMKK9-MPK19-WRKY20 may constitute a signaling module to regulate responses to endogeneous and external stimuli.

As MAPKs are recognized as nodal points in many signaling pathways, further efforts should include identification of their direct substrates and systematic observations of MAPK signaling regulation in specific environmental or developmental contexts. In Arabidopsis, only a few of the 10 MKKs and 20 MPKs as well as their targets have been characterized *in vivo*. For instance, overexpression of AtMKK7 and AtMKK9 accelerate cell death in *N. benthamiana*[57] and AtMPK5 may affect synergism of the JA and SA signaling networks [59]. Since AtWRKY53 regulates leaf senescence and defense against *P. syringae*[60,61] and AtMKK9 was reported to be involved in the biosynthesis of ethylene and camlexin, and also participates in the process of leaf senescence [62,63], we hypothesized that the BnaMKK9-BnaMPK1/2-BnaWRKY53 module we identified in canola may function in leaf senescence, hormone signaling and defense responses. However, although AtMKK9 regulates ethylene signaling through interactions with MPK3 and -6 [64], we did not indentify any interaction of BnaMKK9 with BnaMPK3 or -6. This may reflect an inherent difference in signaling pathways between Arabidopsis and canola, and highlights the necessity to study the MAPK-mediated signaling pathways in canola separately, instead of simply inferring these networks from results in Arabidopsis.

On the other hand, BnaMPK3 and -6 interacted significantly with both upstream BnaMKK2, -4 and -5 and with downstream BnaWRKY20, -26 as shown from the titration and X-gal assays (Figure 6B, C and D). To confirm the authenticity of the interactions between MKK4/5 and MPK3/6, we alternated the Y2H vectors for *MKK4/5* and *MPK3/6*, which is to say that *BnaMKK4/5* were cloned into pGBKT7 and *BnaMPK3/6* were cloned into pGADT7 vector. The interactions between BnaMKK4/5 and BnaMPK3/6 were maintained even after the vectors were switched (data not shown). Similarly, in Arabidopsis, MKK4 interacts with MPK3/6 and MKK5 with MPK6 in Y2H [56]. Moreover, in protein microarrays, Arabidopsis MKK4 interacts with MPK6, and MKK5 with MPK3/6 [57], although these interactions need to be confirmed using a complementary technique, and their biological significance elucidated. Further comparisons revealed that AtMKK2 was reported to interact strongly with MPK4, -6, -10 and -11 but weakly with MPK13, and no interaction between AtMKK2 and MPK3 was detected [56]. Hence, Y2H analysis of interactions between BnaMKK4/5 and BnaMPK3/6 are consistent with previous results with Arabidopsis orthologs. However, the interactions between MKK2 and MPK3/6 were different, despite the relatively close evolutionary relationship between Arabidopsis and canola.

As for the possible signaling components downstream of MPK3 and -6 in Arabidopsis, the function of the module of MEKK1-MKK4/5-MPK3/6 and their downstream targets WRKY22/29 are well known in regulating plant immune responses against *P. syringae*[7]. Another study showed that AtMPK3/MPK6 activation could impede the infection of the fungal pathogen *Botrytis cinerea* while the resistance was compromised in *mpk3* and *mpk6* mutants and, AtMPK3 and 6 function upstream of *Phytoalexin Deficient* (*PAD*) *2* and *-3* to regulate the biosynthesis of camalexin and hence the plant immune response [19]. In Arabidopsis plants expressing constitutively active MKK2 (MKK2-EE), following challenge with *P. syringae*, the levels of JA, ET and SA were elevated rapidly, and the infection was therefore impeded. The downstream targets of MKK2 are MPK4 and 6, and their transcript levels were also increased and the activity of MPK4 was triggered in MKK2-EE infected with *P. syringae*[65]. However, whether BnaMPK3 and -6 and their associated complicated linear MPK pathway function in regulating plant immune response and other processes needs to be further analyzed.

As one group of the direct targets of MAP kinases in plants, WRKY TFs play very important roles in modulating abiotic and biotic stresses responses. One of our previous reports has identified 46 WRKY TF genes from canola and partially characterized some of them in the context of hormone responses and fungal pathogen challenge [44]. To test the interactions between the 12 BnaMPKs and nine BnaWRKYs, we used Y2H. The results demonstrated that BnaMPK3/6 interacted strongly with BnaWRKY20/26 (Figure 6B, C). BnaWRKY20 and BnaWRKY26 belong to Group I of the WRKY gene family. We also found that the amino acid sequences of both BnaWRKY20 and -26 have proline-directed serines (SP cluster) and a docking domain (D domain) at their N-termini, which are widely conserved in group I WRKYs [14]. The SP clusters are putative phosphorylation targets of MAPKs and the D domain is a cluster of basic residues followed by LxL motif ((K/R)_1-2_- × _2-6_-L/I- × -L/I) and it is thought to select the interacting MAPKs and determine the substrate specificity of MAPKs in mammals and yeast [66], however the significance in plants still await to be tested. The identification of two upstream MAP kinases, BnaMPK3 and BnaMPK6, would provide a clue that activities of BnaWRKY20 and -26 are likely regulated by them. So far, the ortholog of BnaWRKY26 in Arabidopsis together with two other Group I members AtWRKY25 and -33 has been shown to regulate salinity responses and thermotolerance [67,68]. However, the upstream regulators, especially MAP kinase(s), of AtWRKY26 in response to either salinity or heat have not been reported. Moreover, the functions of WRKY20 and WRKY26 in relation to signaling in other stresses have not yet been reported. Finally, for some of the nine BnaWRKYs screened, we did not detect any interacting BnaMPKs, which could be due to at least two causes. One possible explanation is that other unidentified BnaMPKs may interact with them. Alternatively, other types of protein kinases, like calcium-dependent protein kinases (CDPKs/CPKs) may act upstream of these BnaWRKYs [69].

Through Y2H, we also experimentally determined that BnaMKK1 interacts with BnaMPK6 (Figure 6D), while in Arabidopsis MKK1 interacts with MPK4/11 only [56], further highlighting the limitations of Arabidopsis as a model for canola. In Arabidopsis, MKK1 and MPK6 both participate in ABA and sugar signaling in the process of seed germination since loss of function mutants of *MKK1*, *MPK6* or both are resistant to ABA or glucose treatments while on the contrary, the overexpression of *AtMKK1* or *AtMPK6* are sensitive to these treatments [22]. Our study of BnaMKK1 interactions with BnaMPK6 presented here suggests that these may play similar roles in seed germination, however these need to be characterized in detail.

**Figure 9:**
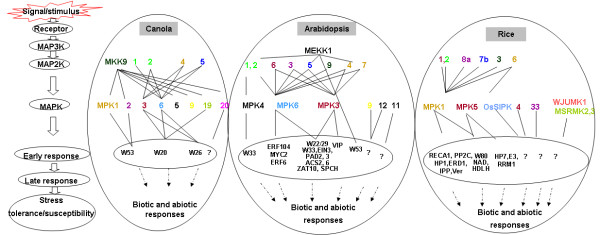
**MKK-MPK mediated responses in canola as compared to those in Arabidopsis and rice.** Orthologs of MKKs or MPKs between three different species are shown in the same colors. Solid lines indicate confirmed interactions as reported in this study and in publications (see the main text and [70]), dashed lines with arrows mean possible regulation at the transcriptional level.

In summary, from our yeast two-hybrid and BiFC assays, two complete signaling modules could be inferred, among which one is BnaMKK9-BnaMPK1/2-BnaWRKY53 and the other BnaMKK2/4/5-BnaMPK3/6-BnaWRKY20/26 (Figure 9). Other MAPK modules have also been identified, with the upstream BnaMKK9 and downstream MAP kinases, BnaMPK5, -9, -19 and -20 (Figure 9). Among these identified interaction pairs, quite a few have not been reported even in Arabidopsis suggesting that the different combination of BnaMKK and BnaMPKs, as well as BnaMPKs and BnaWRKYs very likely mediate the responses to different external and internal stimuli in canola.

### Transactivation and truncation assays of BnaMPK-interacting BnaWRKY transcription factors

To further characterize the biological significance of the aforementioned BnaMPK-interacting BnaWRKY TFs, we performed transactivation assays of BnaWRKY20, -26 and -53 in yeast (Figure 7). We cloned BnaWRKY genes into the vector pGBKT7. The results showed that neither BnaWRKY20 (Figure 7A) nor BnaWRKY26 (data not shown), showed transactivation activity, suggesting that they may be transcriptional repressors. In contrast, BnaWRKY53 showed transactivation activity in yeast (Figure 7C), indicating it is a transcriptional activator. Sequence analysis of the BnaWRKY53 protein sequence showed that it contained a transactivation domain (TAD) near it N-terminus (Figure 7C), while no such domain was detected in BnaWRKY20 (Figure 7A) or -26. Although yeast colonies expressing full length BnaWRKY20 protein could not grow on the selective medium SD-LTHA, colonies expressing a truncated version of BnaWRKY20, namely BnaWRKY20-D1, in which the C-terminal amino acids between 238 and 532 were deleted, grew on SD-LTHA (Figure 7A), suggesting that the sequence of amino acids between 238 and 532 of BnaWRKY20 has the transcriptional repression activity. Interestingly, we also found that BnaWRKY20 can interact with itself in yeast, indicating that it may homodimerize as part of its normal function (Figure 7B). A previous study also demonstrated that another three Arabidopsis WRKY TFs, WRKY18, -40 and -60, which belong to Group IIa, are transcriptional repressors and interact with each other in yeast [71].

To further identify the interacting regions of BnaWRKY20 with BnaMPK3, -6 and -19, we constructed a series of truncated versions of BnaWRKY20 and analyzed their interaction with BnaMPK3, -6 and -19. As shown in Figure 7B, yeast colonies expressing full length of BnaWRKY20 and BnaMPK3, or -6, -19 could grow on selective media SD-LTHA, and yeast colonies expressing BnaWRKY20-D2, BnaMPK3 or -6 could also grow on selection medium SD-LTHA. These results imply that both BnaMPK3 and -6 can interact with both BnaWRKY20 and truncated BnaWRKY20 containing the N-terminal 255 amino acids. As for BnaMPK19, it can only interact with the full length and the C-terminal peptide (amino acids of 231 to 532) of BnaWRKY20 (Figure 7C). For *BnaWRKY53*, we also constructed three deletion plasmids and, Y2H assay demonstrated that both BnaMPK1 and BnaMPK2 interacted with BnaWRKY53-D3, which harbors amino acids of 51-317 (Figure 7C), but did not interact with BnaWRKY53-D1 or BnaWRKY53-D2 (data not shown).

### Expression analyses of BnaMKK and BnaMPK genes in response to abiotic and biotic stresses

Accumulating evidence has demonstrated that members of MKK and MPK gene families in Arabidopsis, rice as well as other species function in responses to abiotic and biotic stresses [2,72]. Therefore, we examined transcript expression profiles of canola BnaMKK and BnaMPK using quantitative real-time RT-PCR (qRT-PCR) under different abiotic, biotic stress treatments as well as hormone stimuli. We treated 18-day-old canola seedlings with jasmonic acid (JA), salicylic acid (SA), abscisic acid (ABA), methyl viologen (MV), ACC, NaCl, cold, oxalic acid (OA) and *S. sclerotiorum*. Among these, JA and SA are two known plant defense hormones, with JA mediating responses to necrotrophs [73] and SA regulating responses to biotrophic pathogens and systemic acquired resistance [74]. ABA is a well-known stress hormone involved in abiotic stress such as salinity and drought. However, recent evidences showed that ABA also has a prominent role in biotic stress [75]. MV was used to induce oxidative burst or accumulation of reactive oxygen species (ROS) in plant cells, which is similar to the effect generated by hydrogen peroxide (H_2_O_2_), but more stable than H_2_O_2_. ACC was used to mock the effect of ethylene, which is an important hormone in many processes including mediating different types of induced resistance [76]. OA is a virulence factor secreted by the necrotrophic pathogen *S. sclerotiorum*[77-79]. Recent studies have shown that controlling OA production might help to protect canola plants against *S. sclerotiorum*[42,80,81].

**Figure 10:**
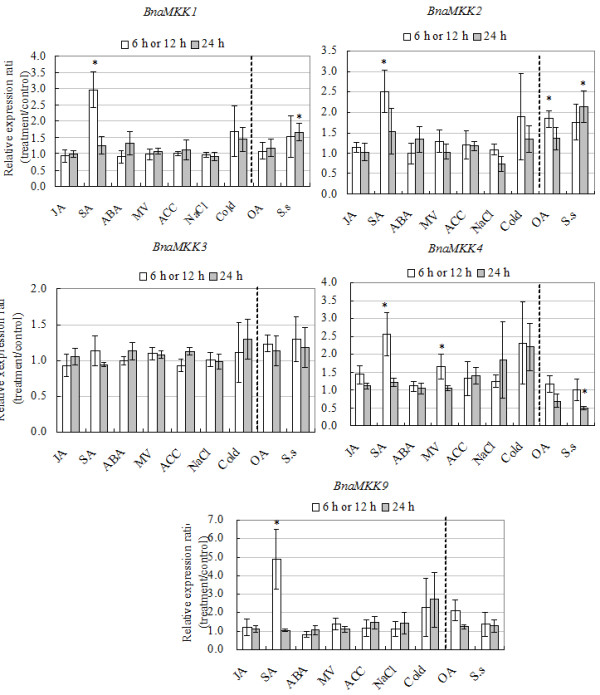
**Expression analyses of BnaMKK genes in response to various treatments, including 20 μM JA, 2 mM SA, 50 μM ABA, 10 μM Paraquat, 1 mM ACC, 200 mM NaCl, cold (4°C), 5 mM oxalic acid and *S. sclerotiorum *infection.** Data is the mean of three biological replicates ± S.E. Asterisks denote significant differences by Student *t*-test analysis (* for p < 0.05 and ** for p < 0.01).

**Figure 11:**
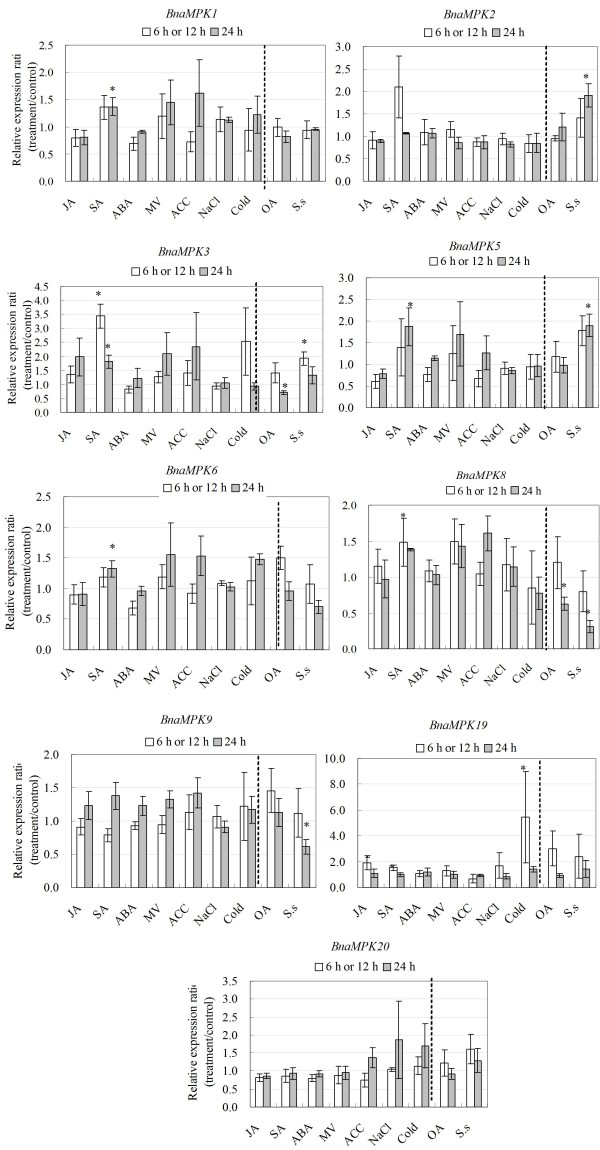
**Expression analyses of BnaMPK genes in response to various treatments, including 20 μM JA, 2 mM SA, 50 μM ABA, 10 μM Paraquat, 1 mM ACC, 200 mM NaCl, cold (4°C), 5 mM oxalic acid and *S. sclerotiorum *infection.** Data is the mean of three biological replicates ± S.E. Asterisks denote significant differences by Student *t*-test analysis (* for p < 0.05 and ** for p < 0.01).

Statistical analyses of qRT-PCR data from three independent biological replicates demonstrated that transcripts of four out of five (80%) BnaMKK genes and six out of nine (67%) BnaMPK genes were significantly induced or repressed by at least one stress condition of cold, OA, SA and *S. sclerotiorum* (Figures 10 and 11). As for BnaMKK genes, four genes (*BnaMKK1*, *-2*, *-4*, and *-9*, 80%) were responsive to SA and only three genes (*BnaMKK1*, *-2*, and *-4*, 40%) were responsive to *S. sclerotiorum*. One gene (*BnaMKK4,* 20%) is significantly induced by MV at 6 h while another gene (*BnaMKK2*, 20%) was responsive to OA. As for BnaMPKs genes, three genes (*BnaMPK3*, *-5*, and *-8*, 33.3%) were responsive to SA, four (*BnaMPK2*, *-5*, *-8* and *-9*, 66.7%) were responsive to *S. sclerotiorum* and, two (*BnaMPK3* and *-8*, 22.2%) were responsive OA treatment (Figure 11). *BnaMPK1, -2, -6* and *-8* were also responsive to SA treatment, although the changes are not significantly (Figure 11, *p* < 0.05). The results we presented here show that *BnaMKK* and *BnaMPK* genes are generally responsive to biotic and abiotic stress treatments and may play an important role in responses to environmental stresses and pathogens. These results also highlight the extent of cross-talk that exists between abiotic and biotic stresses.

### Comparison of the expression patterns of *BnaMKKs* and *BnaMPKs* with those of orthologs in Arabidopsis

To further compare the expression profiles and roles of *BnaMKK* and *BnaMPK* with their orthologs in Arabidopsis, we extracted the expression profiles of AtMKK and AtMPK genes from Genevestigator (https://www.genevestigator.com/gv/plant.jsp). We found that on the whole, the responses of *MKK* and *MPK* genes between canola and Arabidopsis plants at the transcript levels to a variety of abiotic and biotic stress treatments were similar and showed a large degree of correlation (Additional file 8: Table S3). For instance, *AtMKK1*, *-2*, *-4*, and *-9* are all induced by SA treatment, which is consistent with our observation on *BnaMKK1*, *-2*, *-4*, and *-9* (Figure 10). *AtMKK9* is induced by JA, SA, ABA, oxidative stress, salt and cold as well as a necrotrophic fungus, *B. cinerea*, which implies that *AtMKK9* may be involved in response to diverse stresses, while its ortholog in canola is induced significantly only by SA and cold (Figure 10), which suggests that AtMKK9 and BnaMKK9 may play different roles in two different plant species. AtMKK9 was reported to be involved in the biosynthesis of ethylene and camlexin, and also participate the process of leaf senescence [62,63]. We also noted that *BnaMPK19* was induced by cold, which is consistent with the expression pattern of its presumed ortholog in Arabidopsis (Figure 10, Additional file 8: Table S3). AtMKK1 and AtMPKK2 with their upstream AtMEKK1 and downstream AtMPK4 function together in regulating plant innate immunity [5]. *BnaMKK3* did not respond to any treatment tested in our project, however, its ortholog AtMKK3 was reported to participate in JA signaling pathway together with AtMPK6 [82].

In summary, after comparing the transcript expression profiles of MKK and MPK genes between canola and Arabidopsis, we found that most, but not all of them show similar responses towards various abiotic and biotic stress treatments. However, some differences between the *BnaMKKs* and *BnaMPKs* and their Arabidopsis counterparts were noticed. The function of some members of the MKK and MPK gene families in stress-related hormone signaling, for instance, *MKK6*, *MPK1*, *-2*, *-5*, *-8*, *-16*, *-17*, *-19* and *-20* even in Arabidopsis still wait to be studied. Furthermore, what is the function of BnaMPK1 and -2 and are they involved in early signaling process leading to leaf senescence modulated by BnaWRKY53? Based on our recent results from a systematic functional analysis of canola MAPKKK genes, we found that BnaMKK9 is a target for at least three different BnaMAPKKKs (Sun et al, manuscript in preparation), suggesting BnaMKK9 may be a convergence point in the MAPK signaling cascade. Therefore, BnaMKK9 may be integrated in certain signaling pathway in canola defense response and this need to be elucidated through a more detailed functional analysis.

**Figure 12:**
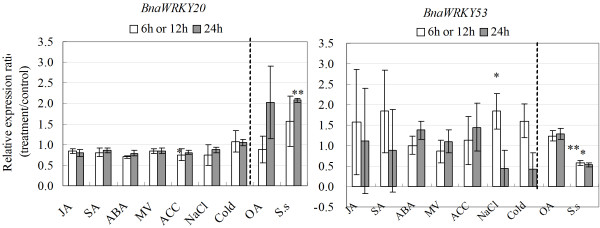
**Transcript expression of BnaWRKY53 in response to various treatments, including 20 μM JA, 2 mM SA, 50 μM ABA, 10 μM Paraquat, 1 mM ACC, 200 mM NaCl, cold (4°C), 5 mM oxalic acid and *S. sclerotiorum *infection.** Data is the mean of three biological replicates ± S.E. Asterisks denote significant differences by Student *t*-test analysis (* for p < 0.05 and ** for p < 0.01).

We were also curious to know whether each component of the linear modules we identified showed similar transcriptional responses to the hormone and stress treatments in a similar trend. We found that components of the same module generally showed similar responses to a given treatment, although some differences were observed. For instance, in the BnaMKK9-BnaMPK5 module, both genes were induced by SA (Figures 10 and 11). In the module of BnaMKK9-BnaMPK1/2-BnaWRKY53, the transcript levels of *BnaMKK9*, *BnaMPK1*, -2 and *BnaWRKY53* were increased by SA while changes in *BnaMPK2* and *BnaWRKY53* were not significant (Figure 10, Additional file 8: Table S3). On the other hand, some components of the same module showed different responses. For instance, in the module of BnaMKK9-BnaMPK5 and BnaMKK9-BnaMPK19, *BnaMKK9* transcripts did not show any response towards *S. sclerotiorum* infection at either 6 or 24 h, while *BnaMPK5* was induced and *BnaMPK19* repressed by *S. sclerotiorum* inoculation (Figure 11). In the module of BnaMKK9-BnaMPK1/2-BnaWRKY53, *BnaMPK2* was induced while *BnaWRKY53* repressed by *S. sclerotiorum* (Figure 12). Our results suggest that *BnaMKK9* may regulate different MPK components in fulfilling different functions in responses to diverse stresses. In addition, in the module of BnaMKK2/4/5-BnaMPK3/6-BnaWRKY20/26, *BnaMKK2, -4*, *BnaMPK3* and *-6* were all induced by SA (Figures 10 and 11), which suggests that this module might be integrated in the SA signaling pathway. Surprisingly, the transcript level changes of each component in this module were different when challenged by *S. sclerotiorum*, *BnaMKK4* was repressed (Figure 11) while *BnaMPK3* and the downstream target *BnaWRKY20* were both induced by *S. sclerotiorum* (Figures 11 and 12). As for modules of BnaMKK1/2-BnaMPK19, we observed that *BnaMKK1* and *-2* were induced by SA, *S. sclerotiorum* and OA (Figure 10) and *BnaMPK19* is induced by cold only (Figure 11). Interestingly, no significant changes on the mRNA abundance of the above selected BnaMKK or BnaMPK genes were observed when JA was applied to seedlings. All of the data suggest that post-transcriptional regulation and post-translational modifications including phosphorylation might play an important role in modulating their kinase activities beyond the transcriptional regulation.

## Conclusions

In the present study, we identified and cloned seven BnaMKK and 12 BnaMPK genes from an important crop, canola (oilseed rape) and reconstructed the phylogeny of both families based on major land plant lineages. Our study complements several previous analyses which inferred the phylogenetics based on limited land plant lineages [1,4,50]. Since the core of MAPK signaling in all eukaryotes is organized in three-tiered modules, it was necessary to dissect the subcellular localization of individual MKK and MAPK members, as well as their interactors to dismantle the complexity of plant MAPK modules and networks. Our GFP-based translational fusions confirmed the cytoplasmic and nuclear subcellular localizations of both BnaMKK and BnaMPK.

Y2H assays and *in vivo* BiFC confirmation identified two linear pathways of MPK signaling modules, composed of BnaMKK-BnaMPK-BnaWRKY and some new interacting partners (Figure 9). The two complete modules inferred based are BnaMKK9-BnaMPK1/2-BnaWRKY53 and BnaMKK2/4/5-BnaMPK3/6-BnaWRKY20/26 (Figure 9). Some incomplete modules were also identified, such as BnaMKK9-BnaMPK5/9/19/20 (Figure 9). Among the identified interaction pairs, many have not been previously identified, even in Arabidopsis. Analysis of their expression patterns suggested that BnaMKK9, BnaMPK1, 2 and BnaMKK2, -4, -5 and BnaMPK3 and -6 may be integrated into the SA signaling pathway to trigger defense response of canola (Figures 10 and 11). Further experiments could be carried on in the protoplast system to accurately determine the linear modules involved signaling pathways. Moreover, genetic analysis of selected MKK and MPK components of canola through overexpression and RNA interference (RNAi) techniques are underway. This work presented here will be helpful for us to better understand the roles of BnaMKK and BnaMPK genes in canola response to environmental stresses, especially the plant response to biotic stresses.

## Methods

### Identification of MKK and MPK genes in *B. napus*

*BnaMKK* and *BnaMPK* gene identification was performed essentially as described previously [44]. Expressed sequence tags (ESTs) representing MKK and MPK genes were retrieved by BLAST searching the NCBI dbEST database (release 110101) using 10 *AtMKKs* and 20 *AtMPKs* as the queries, respectively. Only hits with e-values lower than 10^-4^ were retrieved. Altogether we obtained 18 and 84 unique ESTs representing *BnaMKK*s and *BnaMPKs*, respectively. These ESTs were further cleaned, clustered, and assembled and, each of the resultant contigs and singlets were reciprocally BLAST-searched against Arabidopsis to find the best hit, which we designated as the putative ortholog. Subsequently, the amino acids were predicted using open reading frames (ORFs) in DNASTAR (DNASTAR Inc.). At this step, we identified the cDNA (mRNA) sequences of a total of eight *BnaMKK* and 18 *BnaMPK*.

### Plant growth and gene cloning

Wild type canola (DH12075) plants were grown in Pindstrup soil mix (Egypt) in a glasshouse with a photoperiod of 16 h light (T5 fluorescent tubes with a light intensity of approximately 90 μE m^-2^ s^-1^)/8 h dark, and a temperature of 22°C for 7 d. RNA was isolated from seedlings using a Plant RNA kit (Omega Bio-Tek, USA). RNA integrity was checked by electrophoresis on an agarose gel and quantified using the NanoDrop 1000 (NanoDrop Technologies, Inc., USA). First-strand cDNA was synthesized from 2.5 μg of total RNA using RevertAid H minus reverse transcriptase (Fermentas) and oligo(dT)18 primers (Fermentas). PCR primers were designed using PrimerSelect (DNAStar Inc.) or Primer 3 (v0.4.0, http://frodo.wi.mit.edu/) (Additional file 9: Table S4). PCR was conducted in a 50 μL final volume including 0.5 μL of cDNA template, 1 × *Pfu* buffer, 200 μM deoxynucleotide triphosphates (dNTPs) (Promega), 400 nM of each primer, and 1 unit of *Pfu* polymerase. The PCR conditions included an initial denaturation at 94°C for 3 min, followed by 35 cycles of 94°C for 30 s, 50°C for 30 s, 72°C for 1 min per kb, with a final extension at 72°C for 8 min. RACE (rapid amplification of cDNA end) was performed as described [44]. PCR products were gel purified using the BioSpin Gel Extraction Kit (Bioer Technology Co., Ltd) and cloned into pJET1.2 vector supplied with the CloneJET PCR cloning kit (Fermentas) and sequenced from the two ends using BigDye reagent in ABI3700 sequencer (Applied BioSystems).

### Phylogenetic tree construction and bioinformatics

The *MKKs* and *MAPKs*/*MPKs* of Arabidopsis and rice (*Oryza sativa* subsp. *japonica*) were downloaded from TAIR10 (http://www.arabidopsis.org) and the rice genome annotation project http://rice.plantbiology.msu.edu/annotation_community_families.shtml[1], respectively. To identify *MKKs* and *MAPKs* from other species, we first aligned the amino acid sequences of 10 AtMKKs and 20 AtMPKs and generated a hidden Markov model (HMM) for each. Next we performed an HMM-based search (http://hmmer.janelia.org/) [83], for similar peptide sequences in the sequenced genomes as presented in Phytozome v9.0 (http://www.phytozome.net/) and also by keyword search in the NCBI database. After that, each retrieved sequence was examined for the conserved MKK and MAPK signature motif sequences. The species searched include *Brachypodium distachyon* (*Bd*); *Capsicum annuum* (*Ca*); *Chlamydomonas reinhartdii* (*Cr*); *Euphorbia esula* (*Ee*); *Gossypium hirsutum* (*Gh*); *Glycine max* (*Gm*); *Hordeum vulgare* (*Hv*); *Ipomoea batatas* (*Lb*); *Malus micromalus* (*Mm*); *Medicago sativa* (*Ms*); *Nicotiana tabacum* (*Nb*); *Ostreococcus tauri* (*Ot*); *Petroselinum crispum* (*Pc*); *Physcomitrella patens* (*Pp*); *Pisum sativum* (*Ps*); *Populus trichocarpa* (*Pt*); *Ricinus communis* (*Rc*); *Selaginella moellendorffii* (*Sm*); *Sorghum bicolor* (*Sb*); *Solanum lycopersicum* (*Sl*); *Saccharum officinarum* (*So*); *Solanum tuberosum* (*St*); *Triticum aestivum* (*Ta*); *Vitis vinifera* (*Vv*) and *Zea mays* (*Zm*).

The phylogenetic trees of both MKKs and MPKs across various species were produced as described previously [44]. First, the predicted amino acid sequences of MKKs or MPKs were aligned using ClustalX1.83. Then, by using the maximum parsimony (MP) algorithm implemented in MEGA5.1 [84], the bootstrap consensus trees were inferred from 500 replicates to represent the inferred evolutionary history. The amino acid sequences of MKKs or MPKs from a variety of species retrieved from online databases were also identified for the reconstruction of the phylogenetic trees.

We analyzed the predicted candidate proteins of BnaMKK and BnaMPK gene families using MEME 4.9.0 (Release date: Wed Oct 3 11:07:26 EST 2012) with the parameters as described previously [47,85]. The maximum number of motifs was set to 10 and the optimum motif width was 6 to 50. For prediction of transactivation domain (TAD) of transcription factors, a 9aa TAD prediction program was used (http://www.at.embnet.org/toolbox/9aatad).

### Subcellular localization and confocal microscopy

The coding regions (CDSs) of BnaMKK2, -*3, -4* and *BnaMPK-3, -5, -6,* and *-9,* were amplified by RT-PCR from the cDNA of canola with primers listed (Additional File 9: Table S4). Both pCsGFPBT (GenBank: DQ370426) binary vector and a modified version, pYJGFP with a Gly-Ala rich peptide linker between CDS and sGFP were used in this study [67]. The modified GFP vector, pYJGFP was made by insertion of multiple cloning sites at *Nco* I site and sequenced to confirm that it was in-frame. These vectors and *BnaMKK* or *BnaMPK* products were digested with corresponding restriction enzymes (Ferments), purified (Bioer) and ligated by T_4_ DNA ligase (Fermentas) before transformed into DH5α. After confirmed by sequencing, these constructs and p19 protein of tomato bushy stunt virus were transferred into *Agrobacterium tumefaciens* GV3101 for infiltrating into the leaves of *N. benthamiana*[86]. P19 can suppress gene silencing in transformed tobacco leaves. AS-media (for 100 ml, 1 ml of 1 M MES-KOH, pH 5.6, 333 μl of 3 M MgCl_2_, 100 μl of 150 mM acetosyringon [in DMSO, stored in aliquots at -20°C]) was freshly made to resuspend transformed agrobacteria. One mililiter of the culture was taken by sterile single-use syringes to inject into the abaxial air space of tobacco leaves. The leaf section near the injection site was taken and 500 mM mannitol was used to treat leaves section for one hour at room temperature to separate the cell wall and protoplast. Observation of GFP signal was performed under a confocal microscope LSM510beta (Carl Zeiss).

### Fungal pathogen inoculation and stress treatments

Wild type canola plants were grown in a greenhouse with a photoperiod of 16 h light /8 h dark for 18 d. *S. sclerotiorum* (SX09-904) was a gift provided by Prof. Lili Huang (Northwest A & F University) and was cultured on Potato Dextrose Agar (PDA) mdium. Agar plugs from the front edge of mycelia were used to inoculate the first and the second true leaves with wounding. Leaves inoculated with agar plugs without mycelia were used as mock control. Tissues were harvested at 6 and 24 h post inoculation (hpi) and flash frozen in liquid nitrogen before stored at -80°C. Jasmonic acid (JA, Sigma-Aldrich, USA), SA (Sigma-Aldrich, USA), Paraquat (MV, Sigma-Aldrich), abscisic acid ((±)-ABA, Invitrogen, USA), 1-aminocyclopropane-1-carboxylic acid (ACC, Sigma-Aldrich), oxalic acid (OA, Sigma-Aldrich) were sprayed onto the leaves with a concentration of 100 μM for JA, 2 mM for SA, 50 μM for ABA, 25 μM for ACC, 10 μM for Paraquat and 40 mM for OA. The preparation of stock solution of JA, SA, and ACC followed our previous procedures [44], while OA was prepared as a 40 mM stock solution as described in [87]. The whole process was repeated three times at independently time and thus three biological replicates were prepared.

### Quantitative RT-PCR (qRT-PCR)

The quantitative reverse transcriptase PCR (qRT-PCR) was performed as described earlier [44] with modifications. Total RNA samples were isolated from treated and non-treated canola leaves using Plant RNA kit (Omega, USA), quantified by NanoDrop (NanoDrop Technologies, Inc.) with the integrity checked on 1% (w/v) agarose gel. RNA samples were transcribed into cDNAs by using RevertAid H minus reverse transcriptase (Fermentas,) and oligo(dT)_18_ primers (Fermentas). Primers used for qRT-PCR were designed using PrimerSelect program of DNASTAR (DNASTAR Inc.) targeting 3’UTR of each genes with amplicon sizes of 75-250 bp (Additional file 9: Table S4). Each primer was reciprocally BLAST against the *B. napus* database in NCBI to eliminate cross-amplification. Primer specificity and amplification efficiency were further checked by running standard curves with melting curves examined. Three independent biological replicates were run and the significance was determined with SPSS software (p < 0.05).

### Yeast two-hybrid assay

Yeast two-hybrid (Y2H) analysis was performed in accordance with the Yeast Protocols Handbook (Clontech, USA). The coding regions of BnaMKK, BnaMPK and BnaWRKY genes were amplified from cDNA fragments of canola by high-fidelity PCR, restricted and cloned into pGBKT7 (*BnaMPKs*) or pGADT7 (*BnaMKKs*, *BnaWRKY*s) vectors, respectively, before transformed into yeast strain AH109 through the lithium acetate method. After selection on media (SD-Leucine-Tryptophan, SD-LT; SD-Leucine-Tryptophan-Histidine + 3’AT, [SD-LTH + 3’AT]; SD-Adenine-Histidine-Leucine-Tryptophan, SD-LTHA), the positive interaction transformants were cultivated into Yeast, peptone, dextrose (YEPD) media for serial dilution. The exponentially grown yeast cells were centrifuged at 3000 *g* at room temperature and adjusted to OD_600_ = 0.5 with sterilized double-distilled water. Later on, it was diluted 1/10, 1/100 and 1/1000. Two microliters of the aforementioned serial diluted yeast cells were spotted onto SD-LT, SD-LTH + 3’AT and SD-LTHA media, grown in 30C for 2-5 days, before photographed.

The colony-lift filter assay was conducted following the instruction in the Yeast Protocols Handbook. The freshly grown colonies on the selection media SD-LTHA were transferred onto sterilized 9 cm filter papers and then flash frozen in liquid nitrogen for 20 seconds. After thawed completely, the filter paper with colonies side up was transferred onto presoaked filter paper in 5 ml of staining buffer (60 mM Na_2_HPO_4,_ 39.8 mM NaH_2_PO_4_, 10 mM KCl, 1 mM MgSO_4_, 0.817 mM 5-bromo-4-chloro-3-indolyl-β-D-galactopyranoside [X-gal] and 38.5 mM β-mercaptomethanol) in a 90 mm petri dish for 8-12 h at 37°C. After that, the reaction was stopped and filter paper dried before photographed.

### *In vivo* bimolecular florescence complementation (BiFC) analysis

For yellow fluorescence protein (YFP)-based BiFC, the coding regions of tested *BnaMPKs* were subcloned into pSPYNE(R)173 and, the coding regions of *BnaMKK* or *BnaWRKY* genes were subcloned into pSPYCE(M) vector [88]. The primers used are listed in Additional file 9: Table S4. After confirmation by sequencing, these constructs and p19 of tomato bushy stunt virus were transferred into *A. tumefaciens* GV3101. Freshly grown agrobacterium cultures were resuspended in a freshly made solution containing 10 mM MES-KOH, pH 5.6, 10 mM MgCl_2_ and 0.15 mM acetosyringone, with OD_600_ adjusted to be 0.7-0.8, before mixed at 1:1:1 ratio, incubated at room temperature for 2-4 h, and infiltrated into the leaves of *N. benthamiana*[89]. At 4 d, the leaf sections near the injection sites were taken and used to examine the YFP signals under a confocal microscope A1R (Nikon, Japan).

## Competing interests

The authors are not aware of any potent competing interests.

## Authors’ contributions

Conceived and designed the experiment: YQJ and BY. Performed the experiments: BY, YQJ, WWL, BJY, ZZ, CL, YS, YZ, MJ, FFW, HZ and BW. Analyzed the data: BY, YQJ, WWL and MKD. Provided material: MKD. Wrote the manuscript: BY, MKD and YQJ. All authors read and approved the manuscript.

## Supplementary Material

Additional file 1: Table S1 BnaMPK and BnaMKK EST summary. Click here for file

Additional file 2: Table S2 MKK and MPK sequences from different species used for phylogenetic analysis. Click here for file

Additional file 3: Figure S1 Phylogenetic analysis of MKKs from a variety of species. Click here for file

Additional file 4: Figure S2 A detailed motif analysis and multiple alignment of canola MKKs. Click here for file

Additional file 5: Figure S3 Phylogenetic analysis of MPKs from a variety of species. Click here for file

Additional file 6: Figure S4 Multiple alignment of MAPK domains of BnaMPKs and selected Arabidopsis, rice, *B. distachyon* and *O. tauri* MAPKs. Click here for file

Additional file 7: Figure S5 A detailed motif analysis and multiple alignment of BnaMPKs. Click here for file

Additional file 8: Table S3 Arabidopsis *MPK* and *MKK* expression profiles in response to different stresses. GENEVESTIGATOR database was used to analyze the gene expression levels. Click here for file

Additional file 9: Table S4 Primers used in this study. Click here for file
