# Supplementary material for: Identification and analysis of MKK and MPK gene families in canola (Brassica napus L.)
Source: BMC Genomics. 2013 Jun 11;14:392. doi: 10.1186/1471-2164-14-392 (PMC3701561; doi:10.1186/1471-2164-14-392)
Supplement: Additional file 3: Figure S1 — Phylogenetic analysis of MKKs from a variety of species. [file 1471-2164-14-392-S3.doc]

Group B

Group A

Group C

Group D

Figure S1. Phylogenetic analysis of mitogen-activated protein kinase kinases (MKKs) from a variety of species. The evolutionary relationship was inferred using the maximum parsimony (MP) method implemented in MEGA5.1 program. An MKK from the marine green alga *Ostreococcus tauri* (*Ot*) was used to root the tree. For clarity of presentation, the respective MKK proteins are depicted by a two to three-letter code denoting the species in combination with numbers or locus ID representing the exact MKK from this species. The green highlighted MKK proteins are from Canola. The analysis involved 90 amino acid sequences. The numbers on the nodes are percentages from a bootstrap analysis of 1000 replicates. There were a total of 1637 positions in the final dataset. The MKK proteins from various species can be divided into four major groups (A to D), as supported by highly significant bootstrap values. At, *A. thaliana;* Bd *,Brachypodium distachyon;* Bna, *Brassica* *napus*; Ca*, C. annuum; Cr, Chlamydomonas reinhartdii;* Ee*, E. esula;* Gm*, G. max;*Gh*, G. hirsutum;* Hv*, H. vulgare;* Ib*, I. batatas;*Mm*, M. micromalus;*Ms*, M. sativa;* Nt*, N. tabacum;*Ot, *O. tauri;*Vv*,,V. vinifera;* Os*,O. sativa;*Pc*,P. crispum;*Ps*, P. sativum;*Pt*, P. trichocarpa;* Pp*, P. patens;* Rc, *R. communis;*Sl*, S. lycopersicum;*So*, S. officinarum;* St, *S. tuberosum;*Sb, [*S. bicolor*](app:ds:Sorghum bicolor)*;*Sm*, S. moellendorffii;*Ta*, T. aestivum;* Zm*, Z. mays.*
