# Supplementary material for: Identification and analysis of MKK and MPK gene families in canola (Brassica napus L.)
Source: BMC Genomics. 2013 Jun 11;14:392. doi: 10.1186/1471-2164-14-392 (PMC3701561; doi:10.1186/1471-2164-14-392)
Supplement: Additional file 4: Figure S2 — A detailed motif analysis and multiple alignment of canola MKKs. [file 1471-2164-14-392-S4.pdf]

a

Motif

1
2
3
4
5
6
7
8
9
10

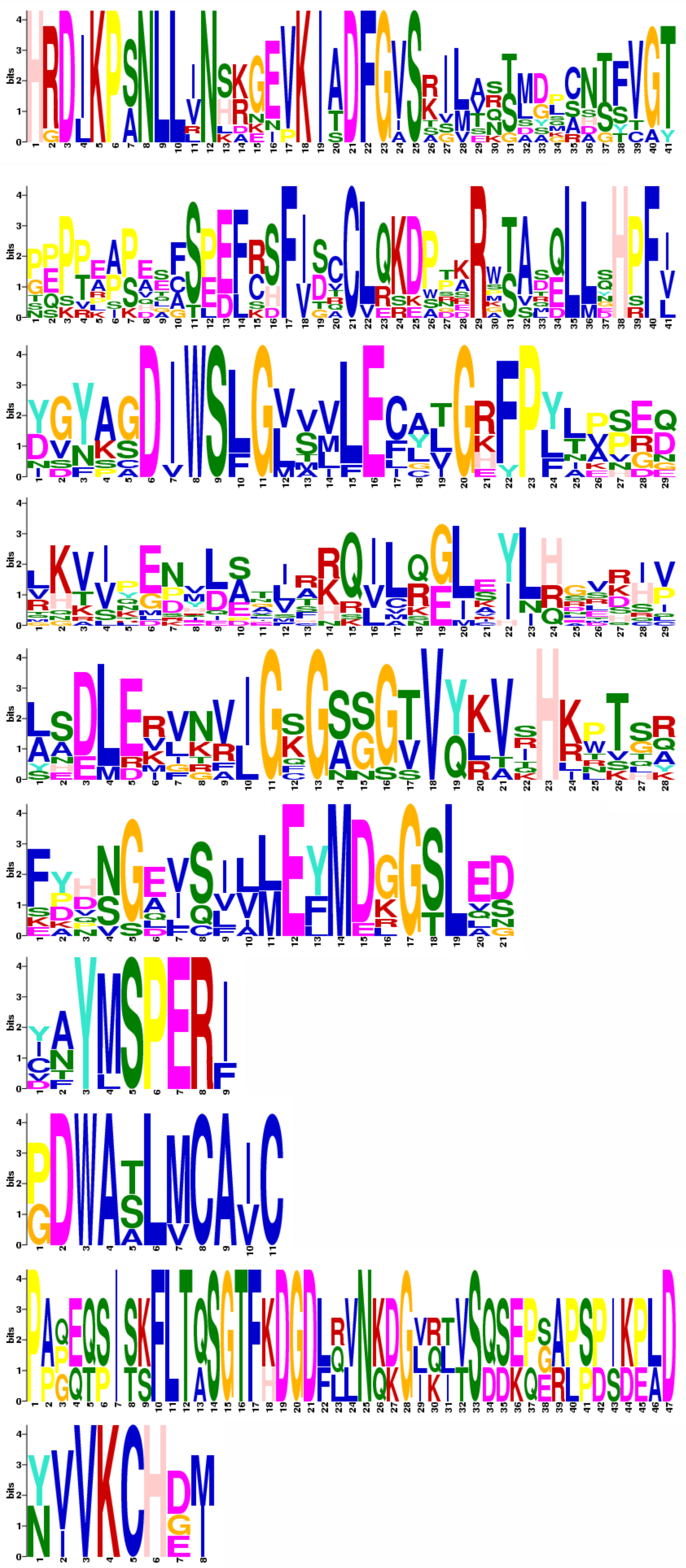

b

```
BnaMKK2     1 -------------MKKGGFSNNLKLSIPPAGEQSITKFLTQSGTFKDGDLRVNKDGVRIV
BnaMKK1     1 -------------MKRGSLSLNP-ISLPPP-EQSISKFLTQSGTFKDGDLQVNKDGIQTV
BnaMKK6     1 -------------MKLKSNLKQLNKLSVPAQQTPISSFLTASGTFHDGDFLLNQKGLKLT
BnaMKK4     1 ------------------MKPVQSAPVKSRPRRRPDLSLPLPQRDVSLAVPLPLPP-TSG
BnaMKK5     1 --------------------------MAMRRNKRPDLSLPLPNRNVALAVPLPLPPPPSS
BnaMKK9     1 ------------------------------------MALVRERRQLNLRLRLPPISDRRF
BnaMKK3     1 MAALEELKKKLSPLFDAEKGFSSSSSLDPNDSYLLSDGGTVNLLSRSYGVYNFNELGLQK
consensus   1              .            ..      ..  ..     .   . .

                                     Motif 5
BnaMKK2    48 SQSEPEALSPIKPADDKLGLSDLDMVKFIGKGSSGVVQLVQHKWTGQFFALKVIQLNVDE
BnaMKK1    46 SQSEPGAPPPIDPLDNQLSLADLEVIKVIGKGSSGSVQLVKHKLTQQFFATKVIQLNTEE
BnaMKK6    48 SDDKQSRPSDSKELDFEITAEDLEIVRVIGKGSGGVVQLVRHKWVGKLFAMKVIQMNIQE
BnaMKK4    42 GSSSSSSSTSSSSSAAPKNYSDLERGNRIGSGAGGTVYKVTHRPSSRVYALKVIYGNHEE
BnaMKK5    35 SSALASSSAISTNISAAKRLSELERVNRIGSGAGGTVYKVIHLPTSRPFALKVIYGNHDD
BnaMKK9    25 SLPSITTAASSTVLSNSISAADLEKLNVLGCGNSGTVYKVSHKNT--LYALKTVNGDMDP
BnaMKK3    61 CTTSLVDESESSETTYQCASHEMRVFGAIGSGASSVVQRAIHIPKHRLLALKRIN-IFER
consensus  61 . .  .  .  . .. .    .    .....   .* *  ...*...  *.  .  ...*.*... .. ..

                                                   Motif 6           Motif 4
BnaMKK2   108 AIHKSIAQELKINQSS-QCPYLVTSYQSFYDN--GAISLILEYMDGGSLEDFLKSVKTIP
BnaMKK1   106 STCRAISQELRINLAS-QCPYLVSCYQSFYHN--GLVSIVMEFMDGGSLLDLLKKVQRVP
BnaMKK6   108 EIRKQIVQELKINQASSQCPHVVVCYHSFYHN--GAFSLVLEYMDRGSLVDVIRQVKTIL
BnaMKK4   102 NVRRQICREIEILRDV-SHQNIVKCHEMFDQ--NGEIQVLLEFMDGGSLEN-RHVSN---
BnaMKK5    95 NVRRQICREIEILRSV-DHPNVVKCHDMFDH--NGEVQVLLEFMDKGSLEG-RHVSR---
BnaMKK9    83 ILTRQLMREMEILRRT-DSPYVVKCHGIFEKPVVGEVSILMEYMDGGTLESLRGAVT---
BnaMKK3   120 EKRQQLLTEIRTLCEAPCHEGLVDFHGAFYSPDSGQISIALEYMDGGSLADILKVTKKIP
consensus 121  .....   *....   .    . .*  ..  *..   *  .....*.**.*.*..... .. .

                     Motif 4                        Motif 1
BnaMKK2   165 ESYLSTIFKQVLQGLIYLHHDKHIIHRDLKPSNLLVNHRGEVKITDFGVSTVMTNTAGLA
BnaMKK1   163 ENMLAAISKRVLRGLCYIHDERRIIHRDLKPSNLLINHRGEVKIADFGVSKILSSTSSLA
BnaMKK6   166 EPYLSVVCKQVLQGLVYLHNERHVIHRDIKPSNLLVNHKGEVKISDFGVSASLASSMGQR
BnaMKK4   155 ERDLAHLSHQILNGLAYLHG-RHIVHRDIKPSNLLINSDKIVKIADFGVSRILAQTMDPC
BnaMKK5   148 ENELAGLTRQILSGLAYIHR-RHIVHRDIKPSNLLINSANNVKIADFGVSRILAQTMDPC
BnaMKK9   139 EKRLAGFARQILKGLSYLHG-LKIVHRDIKPANLLLNSKGEVKIADFGVSKILVRSLDSC
BnaMKK3   180 EPVLSSMFHKLLQGLSYLHGVRHLVHRDIKPANLLVNLKGEPKITDFGISSGLENSMAMC
consensus 181 *  *. . ...*.** *.*  ....***.**.***.*  ....**.***.*... ...  .

              Motif 1  Motif 7                 Motif 3
BnaMKK2   225 NTFVGTYNYMSP---ERI-----VGNKYGNKSDIWSLGLVVLECATGKFPYLPPDEEETW
BnaMKK1   223 HTFVGTDFYMSP---ERI-----SGKAYGNKCDIWSLGVVLLECATGKFPYTPPENMKGW
BnaMKK6   226 DTFVGTYNYLSP---ERI-----SGSTYDYSSDIWSLGMSVLECAIGRFPYLESEDQQNP
BnaMKK4   214 NSSVGTIAYMSP---ERINTDLNQGRYDGYAGDIWSLGVSILEFYLGRFPFNVSRQG---
BnaMKK5   207 NSSVGTIAYMSP---ERINTDLNHGRYDGYAGDIWSLGVSVLEFYLGRFPFAVSRQG---
BnaMKK9   198 NSYVGTCAYMSP---ERFDSGG--GSSDVYAGDIWSFGLMMLELLVGHFPLLPPGQRP--
BnaMKK3   240 ATFVGTVTYMSPERIR--------NDGYSYPADIWSLGLALFECGTGEFPYIANEGP---
consensus 241 ...***  *.**   ...       .  ... .****.*. ..*...*.**..  ..

                                          Motif 2
BnaMKK2   277 SSVFELMEAIVDQPPPTLPSESFSPELSSFISTCLQKDPNSRSSARELMEHPFVKKYDNN
BnaMKK1   275 TNMYELVDAIVENPPPRAPSHLFSPEFCSFISQCVQKDPRDRKSAMELLDHRFVNMFED-
BnaMKK6   278 PSFYELLAAIVESPPPTAPSDQFSPEFCSFITACLQKDPPARASSLDLLSHSFIKKFED-
BnaMKK4   268 -DWASLMCAICMSQPPEAP-AAASPDFRHFISCCLQREPGRRMTATQLLQHPFIRRAVSQ
BnaMKK5   261 -DWASLMCAICMTQPPEAP-ATASEDFRHFISCCLQSDPPKRWSAQQLLQHPFIVKSSGG
BnaMKK9   251 -DWATLMCAVCFGEPPRAP-EGCSEEFRSFVDCCLRKDSTKRWTASQLLGHPFLQEEL--
BnaMKK3   289 ---VNLMLQILDDPSPTPSKQEFSPEFCSFIDVCLQKDADARPTADQLLSHPFITKHEK-
consensus 301    .  *. .... ..* ..    .*... .*.. *.....   * .. .*. *.*. . .
```

```
BnaMKK2    337 SEINLASYFTNAGSPLATLKNLSGTFSV--------------------------------
BnaMKK1    334 VDVDLSSYFTAAGSLIPPLANS--------------------------------------
BnaMKK6    337 MDIDLGILVGTLEPPVNCLR----------------------------------------
BnaMKK4    326 GLNRSPQNLHQLLPPPPPRTLSSSSSSSPTT-----------------------------
BnaMKK5    319 ------PNLRQMLPPPPPAS----------------------------------------
BnaMKK9        ------------------------------------------------------------
BnaMKK3    345 ESVDLAAFVRSIFDPTQRLKDIADMLTIHYYSLFDGYDDLWHHARSLYTETSVFSFSGKQ
consensus  361   . .   .  .  .   .

BnaMKK2        ------------------------------------------------------------
BnaMKK1        ------------------------------------------------------------
BnaMKK6        ------------------------------------------------------------
BnaMKK4        ------------------------------------------------------------
BnaMKK5        ------------------------------------------------------------
BnaMKK9        ------------------------------------------------------------
BnaMKK3    405 YTGSTEILSQLSNIRNTLAGDLPSEKLVHVVEKLQCKPHGVGGVMIRAIGSFIVGNQFLI
consensus  421

BnaMKK2        ------------------------------------------------------
BnaMKK1        ------------------------------------------------------
BnaMKK6        ------------------------------------------------------
BnaMKK4        ------------------------------------------------------
BnaMKK5        ------------------------------------------------------
BnaMKK9        ------------------------------------------------------
BnaMKK3    465 CGDGVQAEGLPSFKDLGFDLGTRRVGRFQEQFVVESGDLIGRYFIAKQELYITS
consensus  481
```

Figure S2. A detailed motif analysis and multiple alignment of canola MKKs.

a, The analysis is from MEME 4.9.0 (Release date: Wed Oct 3 11:07:26 EST 2012).
Stack heights represent conservation at a position, and symbol heights within a stack represent the relative frequency of each residue.
b, Multiple alignment of BnaMKKs with 7 motifs shown as overbars.
