# Supplementary material for: Identification and analysis of MKK and MPK gene families in canola (Brassica napus L.)
Source: BMC Genomics. 2013 Jun 11;14:392. doi: 10.1186/1471-2164-14-392 (PMC3701561; doi:10.1186/1471-2164-14-392)
Supplement: Additional file 5: Figure S3 — Phylogenetic analysis of MPKs from a variety of species. [file 1471-2164-14-392-S5.doc]

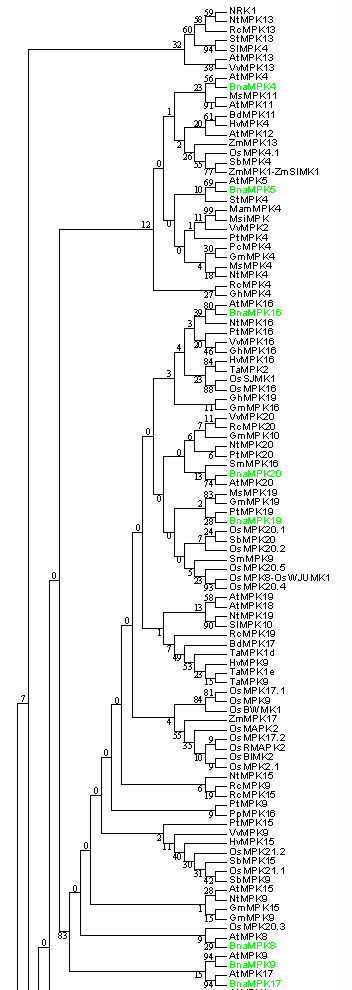


Group B

TEY or MEY motif

Group D

TDY or MEY motif


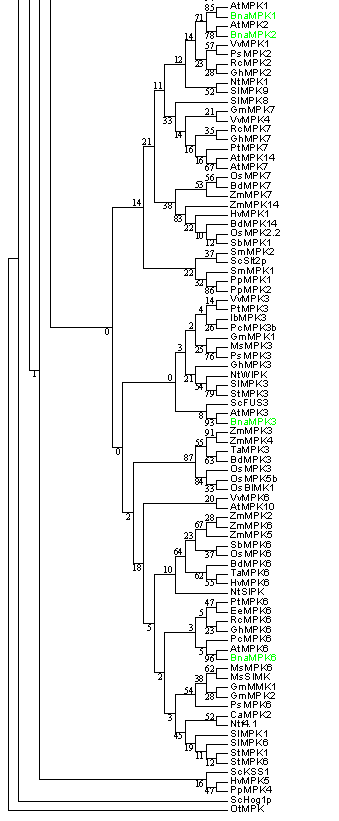


Group C

TEY motif

Group A

TEY motif

Figure S3. Phylogenetic analysis of mitogen-activated protein kinases (MAPKs/MPKs) from a variety of species. The evolutionary relationship was inferred using the maximum parsimony (MP) method implemented in MEGA5.1 program. An MPK from the marine green alga *Ostreococcus tauri* (*Ot*) was used to root the tree. For clarity of presentation, the respective MPK proteins are depicted by a two to three-letter code denoting the species in combination with numbers representing the exact MPK from this species. The MPK proteins from various species can be divided into four major groups (A to D), as supported by highly significant bootstrap values and, the phosphorylation motifs (TEY, TDY or MEY) are different from one group to another. The atypical MEY motif in the activation loop of some MPKs is marked by arrows. The green highlighted MPK proteins are from Canola. The analysis involved 197 amino acid sequences. The numbers on the nodes are percentages from a bootstrap analysis of 500 replicates. There were a total of 850 positions in the final dataset. At, *A. thaliana;* Bd *,Brachypodium distachyon;* Bna, *Brassica* *napus*; Ca*, C. annuum;* Ee*, E. esula;* Gm*, G. max;*Gh*, G. hirsutum;* Hv*, H. vulgare;* Ib*, I. batatas;*Mm*, M. micromalus;*Ms*, M. sativa;* Nt*, N. tabacum;*Ot, *O. tauri;*Vv*,,V. vinifera;* Os*,O. sativa;*Pc*,P. crispum;*Ps*, P. sativum;*Pt*, P. trichocarpa;* Pp*, P. patens;* Rc, *R. communis;*Sl*, S. lycopersicum;*So*, S. officinarum;* St, *S. tuberosum;*Sb, [*S. bicolor*](app:ds:Sorghum bicolor)*;*Sm*, S. moellendorffii;*Ta*, T. aestivum;* Zm*, Z. mays.*
