# Supplementary material for: Identification and analysis of MKK and MPK gene families in canola (Brassica napus L.)
Source: BMC Genomics. 2013 Jun 11;14:392. doi: 10.1186/1471-2164-14-392 (PMC3701561; doi:10.1186/1471-2164-14-392)
Supplement: Additional file 6: Figure S4 — Multiple alignment of MAPK domains of BnaMPKs and selected Arabidopsis, rice, B. distachyon and O. tauri MAPKs. [file 1471-2164-14-392-S6.pdf]

```
BnaMPK4      80   FDNIIDAKRTLREIKLLKHMDHENVIAVKDIIRPPLRENFNDVYIVYELMDTDLHQIIRS
AtMPK4       80   FDNIIDAKRTLREIKLLKHMDHENVIAVKDIIKPPQRENFNDVYIVYELMDTDLHQIIRS
AtMPK11      77   FGNIIDAKRTLREIKLLKHMDHDNVIAIIDIIRPPQPDNFNDVHIVYELMDTDLHHIIRS
AtMPK12      78   FDNIIDAKRTLREIKLLRHMDHENVITIKDIVRPPQRDIFNDVYIVYELMDTDLQRILRS
BdMPK11      91   FDNQIDAKRTLREVKLLRHMNHENVISIKDIIRPPRRENFNDVYIVYELMDTDLHHLLRS
OsMPK4       80   FDNHIDAKRTLREIKLLRHMDHENIIAIKDIIRPPRRDNFNDVYIVSELMDTDLHQIIRS
BnaMPK5      77   FDNKVDAKRTLREIKLLRHLEHENVVIIKDIIRPPKKETFTDVYIVYELMDTDLHQIIRS
AtMPK5       79   FDNKVDAKRTLREIKLLRHLEHENVVVIKDIIRPPKKEDFVDVYIVFELMDTDLHQIIRS
AtMPK13      70   FDNRVDAKRTLREIKLLSHMDHDNVIKIKDIIELPEKERFEDVYIVYELMDTDLHQIIRS
BnaMPK3      75   FDNHMDAKRTLREIKLLRHLDHENIIAIRDVVPPPLRRQFSDVYIATELMDTDLHQIIRS
AtMPK3       75   FDNHMDAKRTLREIKLLRHLDHENIIAIRDVVPPPLRRQFSDVYISTELMDTDLHQIIRS
OsMPK3       73   FNNDMDAKRTLREIKLLRHLDHENIIGIRDVIPPPIPQAFNDVYIATELMDTDLHHIIRS
BdMPK3       73   FDNNMDAKRTLREIKLLRHLDHENIVGLRDVIPPAIPQSFNDVYIATELMDTDLHHIIRS
BnaMPK6      97   FDNKIDAKRTLREIKLLRHMDHENIVAIRDIIPPPLRTAFNDVYIAYELMDTDLHQIIRS
AtMPK6       100  FDNKIDAKRTLREIKLLRHMDHENIVAIRDIIPPPLRNAFNDVYIAYELMDTDLHQIIRS
OsMPK6       104  FDNKIDAKRTLREIKLLRHMDHENIVAIRDIIPPPQRNSFNDVYIAYELMDTDLHQIIRS
BdMPK6       97   FDNKIDAKRTLREIKLLRHMDHENIVAIRDIIPPAQRNSFNDVYIAYELMDTDLHQIIRS
AtMPK10      98   FDNTIEAKRTLREIKLLRHFDHENIVAIRDVILPPQRDSFEDVYIVNELMEFDLYRTLKS
OtMPK        138  FENVVDAKRTLREIKLLRHLRHENVIDIIDCVRPEAMDAFEDVYLMYDLMDTDLYQIIRS
BnaMPK1      69   YENRIDALRTLRELKLLRHLRHENVIALKDVMMPIHKRSFKDVYLVYELMDTDLHQIIKS
AtMPK1       69   YENRIDALRTLRELKLLRHLRHENVIALKDVMMPIHKMSFKDVYLVYELMDTDLHQIIKS
BnaMPK2      69   FQNRIDALRTLRELKLLRHLRHDNVIALKDVMMANHKRTFKDVYLVYELMDTDLHQIIKS
AtMPK2       69   FENRIDALRTLRELKLLRHLRHENVVALKDVMMANHKRSFKDVYLVYELMDTDLHQIIKS
OsMPK2       69   FDNRVDALRTLRELKLLRHLRHENVIALKDIMMPVHRRSFKDVYLVYELMDTDLHQIIKS
OsMPK14      69   FDNRVDALRTLRELKLLRHLRHENVIALKDIMMPVHRRSFKDVYLVYELMDTDLHQIIKS
BdMPK14      69   FDNRVDALRTLRELKLLRHLRHENVISLKDIMMPVQRRSFKDVYLVYELMDTDLHQIIKS
OsMPK7       69   FDNRVDALRTLRELKLLRHLRHENVIALKDIMMPVHRRSFKDVYLVYELMDTDLHQIIKS
BdMPK7       113  FDNRVDALRTLRELKLLRHLRHENVIALKDIMMPVHRRSFKDVYLVSELMDTDLHQIIKS
AtMPK7       69   FENRVDALRTLRELKLLRHVRHENVIALKDVMLPANRSSFKDVYLVYELMDTDLHQIIKS
AtMPK14      69   FENRIDALRTLRELKLLRHVRHENVISLKDVMLPTHRYSFRDVYLVYELMDSDLNQIIKS
AtMPK19      62   FEHVSDALRILREVKLLRLLRHPDIVEIKSIMLPPSKREFKDIYVVFELMESDLHQVIKA
BnaMPK19     62   FEHISDALRILREVKLLRLLRHPDIVEIKSIMLPPSKREFKDIYVVFELMESDLHQVIKA
AtMPK18      62   FEHISDALRILREVKLLRLLRHPDIVEIKSIMLPPSKREFKDIYVVFELMESDLHQVIKA
BnaMPK20     73   FEHISDAARILREIKLLRLLRHPDIVEIKHIMLPPSRREFKDIYVVFELMESDLHQVIKA
AtMPK20      72   FEHISDAARILREIKLLRLLRHPDIVEIKHIMLPPSRREFKDIYVVFELMESDLHQVIKA
OsMPK20      72   FEHISDAARILREIKLLRLLRHPDIVEIKHIMLPPSRRDFKDIYVVFELMESDLHQVIKA
AtMPK8       141  FEHVSDATRILREIKLLRLLRHPDVVEIKHIMLPPSRREFRDIYVVFELMESDLHQVIKA
BnaMPK8      132  FEHVSDATRILREIKLLRLLRHPDVVEIKHIMLPPSRREFRDIYVVFELMESDLHQVIKA
BnaMPK9      60   FEHVSDATRILREIKLLRLLRHPDIVEIKHVMLPPSRREFRDIYVVFELMESDLHQVIKA
AtMPK9       60   FEHVSDATRILREIKLLRLLRHPDIVEIKHVMLPPSRREFRDIYVVFELMESDLHQVIKA
OsMPK17      124  FEHVSDATRILREIKLLRLLRHPDIVEIKHIMLPPSRREFQDIYVVFELMESDLHQVIRA
BdMPK17      124  FEHVSDATRILREVKLLRLLRHPDVVEIKHIMLPPSRREFQDIYVVFELMESDLHQVIRA
BnaMPK16     50   FEHVSDATRILREIKLLRLLRHPDIVEIKHILLPPSRREFRDIYVVFELMESDLHQVIKA
AtMPK16      62   FEHVSDATRILREIKLLRLLRHPDIVEIKHILLPPSRREFRDIYVVFELMESDLHQVIKA
OsMPK16      50   FEHVSDATRILREIKLLRLLRHPDIVEIKHILLPPSRREFKDIYVVFELMESDLHQVIKA
OsMPK21      142  FDHVSDATRILREIKLLRLLRHPDIVEIKHIMLPPSRREFRDIYVIFELMESDLHQVIKA
AtMPK15      127  FDHISDATRILREIKLLRLLLHPDVVEIKHIMLPPSRREFRDVYVVFELMESDLHQVIKA
BnaMPK17     53   FEHVSDAIRILREIKLLRLLKHPDIVEIKHIMLPPCRKEFKDIYVVFELMESDLHHVLKV
AtMPK17      53   FEHVSDAIRILREIKLLRLLRHPDIVEIKHIMLPPCRKEFKDIYVVFELMESDLHHVLKV
consensus         fdn idA RtLREiKLLrhlrHenvv ikdimlpp rr FkDvyivyeLMdtDLhqiiks
```

```
BnaMPK4      139 NQPLTDDHCRFFLYQLLRGLKYVHSANVLHRDLKPSNLLLNANCDLKLGDFGLART----
AtMPK4       139 NQPLTDDHCRFFLYQLLRGLKYVHSANVLHRDLKPSNLLLNANCDLKLGDFGLART----
AtMPK11      136 NQPLTDDHSRFFLYQLLRGLKYVHSANVLHRDLKPSNLLLNANCDLKIGDFGLART----
AtMPK12      137 NQTLTSDQCRFLVYQLLRGLKYVHSANILHRDLRPSNVLLNSKNELKIGDFGLART----
BdMPK11      150 NQPLTDDHCQYFLYQVLRGLKYVHSANVLHRDLRPSNLLLNAKCDLKIGDFGLART----
OsMPK4       139 NQPLTDDHCQYFLYQLLRGLKYVHSANVLHRDLKPSNLFLNANCDLKIADFGLART----
BnaMPK5      136 DQPLTDDHCQYFLYQILRGLKYIHSANVLHRDLKPSNLLLNANCDLKIADFGLART----
AtMPK5       138 NQSLNDDHCQYFLYQILRGLKYIHSANVLHRDLKPSNLLLNSNCDLKITDFGLART----
AtMPK13      129 TQTLTDDHCQYFLYQILRGLKYIHSANVLHRDLKPSNLVLNTNCDLKICDFGLART----
BnaMPK3      134 NQGLSEEHCQYFLYQLLRGLKYIHSAKVIHRDLKPSNLLLNANCDLKICDFGLARP----
AtMPK3       134 NQSLSEEHCQYFLYQLLRGLKYIHSANIIHRDLKPSNLLLNANCDLKICDFGLARP----
OsMPK3       132 NQELSEEHCQYFLYQILRGLKYIHSANVIHRDLKPSNLLLNANCDLKICDFGLARP----
BdMPK3       132 NQELSEEHCQYFLYQLLRGLKYIHSANVIHRDLKPSNLLLNANCDLKICDFGLARP----
BnaMPK6      156 NQGLSEEHCQYFLYQILRGLKYIHSANVLHRDLKPSNLLLNANCDLKICDFGLARV----
AtMPK6       159 NQALSEEHCQYFLYQILRGLKYIHSANVLHRDLKPSNLLLNANCDLKICDFGLARV----
OsMPK6       163 NQALSEEHCQYFLYQILRGLKYIHSANVLHRDLKPSNLLLNANCDLKICDFGLART----
BdMPK6       156 NQALSEEHCQYFLYQILRGLKYIHSANVLHRDLKPSNLLLNANCDLKICDFGLART----
AtMPK10      157 DQELTKDHGMYFMYQILRGLKYIHSANVLHRDLKPSNLLLSTQCDLKICDFGLARA----
OtMPK        197 SQSLTDEHCQYFLYQILRGLKYIHSADVLHRDLKPGNLLLNANCDLKICDFGLARTALVD
BnaMPK1      128 SQVLSNDHCQYFLFQLLRGLKYIHSANILHRDLKPGNLLINANCDLKICDFGLARTS---
AtMPK1       128 SQVLSNDHCQYFLFQLLRGLKYIHSANILHRDLKPGNLLVNANCDLKICDFGLARAS---
BnaMPK2      128 SQVLSNDHCQYFLFQLLRGLKYIHSANILHRDLKPGNLLVNANCDLKICDFGLARMS---
AtMPK2       128 SQVLSNDHCQYFLFQLLRGLKYIHSANILHRDLKPGNLLVNANCDLKICDFGLARTS---
OsMPK2       128 PQGLSNDHCQYFLFQLLRGLKYLHSAEILHRDLKPGNLLVNANCDLKICDFGLARTN---
OsMPK14      128 PQGLSNDHCQYFLFQLLRGLKYLHSAEILHRDLKPGNLLVNANCDLKICDFGLARTN---
BdMPK14      128 PQGLSNDHCQYFLFQLLRGLKYLHSAEILHRDLKPGNLLVNANCDLKICDFGLARTN---
OsMPK7       128 SQPLSNDHCQYFLFQLLRGLKYLHSAGILHRDLKPGNLLVNANCDLKICDFGLARTN---
BdMPK7       172 SQPLSNDHCQYFLFQLLRGLKYLHSAGILHRDLKPGNLLVNANCDLKICDFGLARTN---
AtMPK7       128 SQSLSDDHCKYFLFQLLRGLKYLHSANILHRDLKPGNLLVNANCDLKICDFGLARTS---
AtMPK14      128 SQSLSDDHCKYFLFQLLRGLKYLHSANILHRDLKPGNLLVNANCDLKICDFGLART----
AtMPK19      121 NDDLTREHHQFFLYQMLRALKYMHTANVYHRDLKPKNILANANCKLKVCDFGLARVSFND
BnaMPK19     121 NDDLTKEHHQFFLYQMLRALKYMHTANVYHRDLKPKNILANANCKLKVCDFGLARVSFND
AtMPK18      121 NDDLTREHHQFFLYQMLRALKFMHTANVYHRDLKPKNILANANCKLKVCDFGLARVAFND
BnaMPK20     132 NDDLTKEHYQFFLYQLLRALKYIHTANVYHRDLKPKNILANANCKLKICDFGLARVAFND
AtMPK20      131 NDDLTREHYQFFLYQLLRALKYIHTANVYHRDLKPKNILANANCKLKICDFGLARVAFND
OsMPK20      131 NDDLTKEHYQFFLYQLLRALKYIHTANVYHRDLKPKNILANSNCKLKICDFGLARVAFND
AtMPK8       200 NDDLTPEHYQFFLYQLLRGLKYVHAANVFHRDLKPKNILANADCKLKICDFGLARVSFND
BnaMPK8      191 NDDLTPDHYQFFLYQLLRGLKYVHAANVFHRDLKPKNILANADCKLKICDFGLARVSFND
BnaMPK9      119 NDDLTPEHYQFFLYQLLRGLKFIHTANVFHRDLKPKNILANSDCKLKICDFGLARVSFND
AtMPK9       119 NDDLTPEHYQFFLYQLLRGLKFIHTANVFHRDLKPKNILANSDCKLKICDFGLARVSFND
OsMPK17      183 NDDLTPEHYQFFLYQLLRALKYIHAANVFHRDLKPKNILANSDCKLKICDFGLARASFND
BdMPK17      183 NDDLTPEHYQFFLYQLLRALKYIHAANVFHRDLKPKNILANSDCKLKICDFGLARVSFND
BnaMPK16     109 NDDLTPEHYQFFLYQLLRGLKYIHTANVFHRDLKPKNILANADCKLKICDFGLARVAFND
AtMPK16      121 NDDLTPEHYQFFLYQLLRGLKYIHTANVFHRDLKPKNILANADCKLKICDFGLARVAFND
OsMPK16      109 NDDLTPEHYQFFLYQLLRGLKYIHTANVFHRDLKPKNILANADCKLKICDFGLARVAFSD
OsMPK21      201 NDDLTPEHHQFFLYQLLRGMKYIHAASVFHRDLKPKNILANADCKLKVCDFGLARVSFND
AtMPK15      186 NDDLTPEHHQFFLYQLLRGLKYVHAANVFHRDLKPKNILANADCKLKICDFGLARVSFND
BnaMPK17     112 NDDLTPQHHQFFLYQLLRGLKFMHSAHVFHRDLKPKNILANADCKIKICDLGLARVSFTD
AtMPK17      112 NDDLTPQHHQFFLYQLLRGLKFMHSAHVFHRDLKPKNILANADCKIKICDLGLARVSFTD
consensus        nq Lt ehcqyflyQlLRglKyiHsAnvlHRDLkP NlllnancdlKicDfGLAR
```

Figure S4. Multiple alignment of MAPK domains of BnaMPKs and selected Arabidopsis, rice, *B. distachyon* and *O. tauri* MAPKs. Alignment was performed using Clustal x1.83 and illustrated with Boxshade. The closed triangles represent highly conserved amino acids.
