# Supplementary material for: Identification and analysis of MKK and MPK gene families in canola (Brassica napus L.)
Source: BMC Genomics. 2013 Jun 11;14:392. doi: 10.1186/1471-2164-14-392 (PMC3701561; doi:10.1186/1471-2164-14-392)
Supplement: Additional file 7: Figure S5 — A detailed motif analysis and multiple alignment of BnaMPKs. [file 1471-2164-14-392-S7.pdf]

a

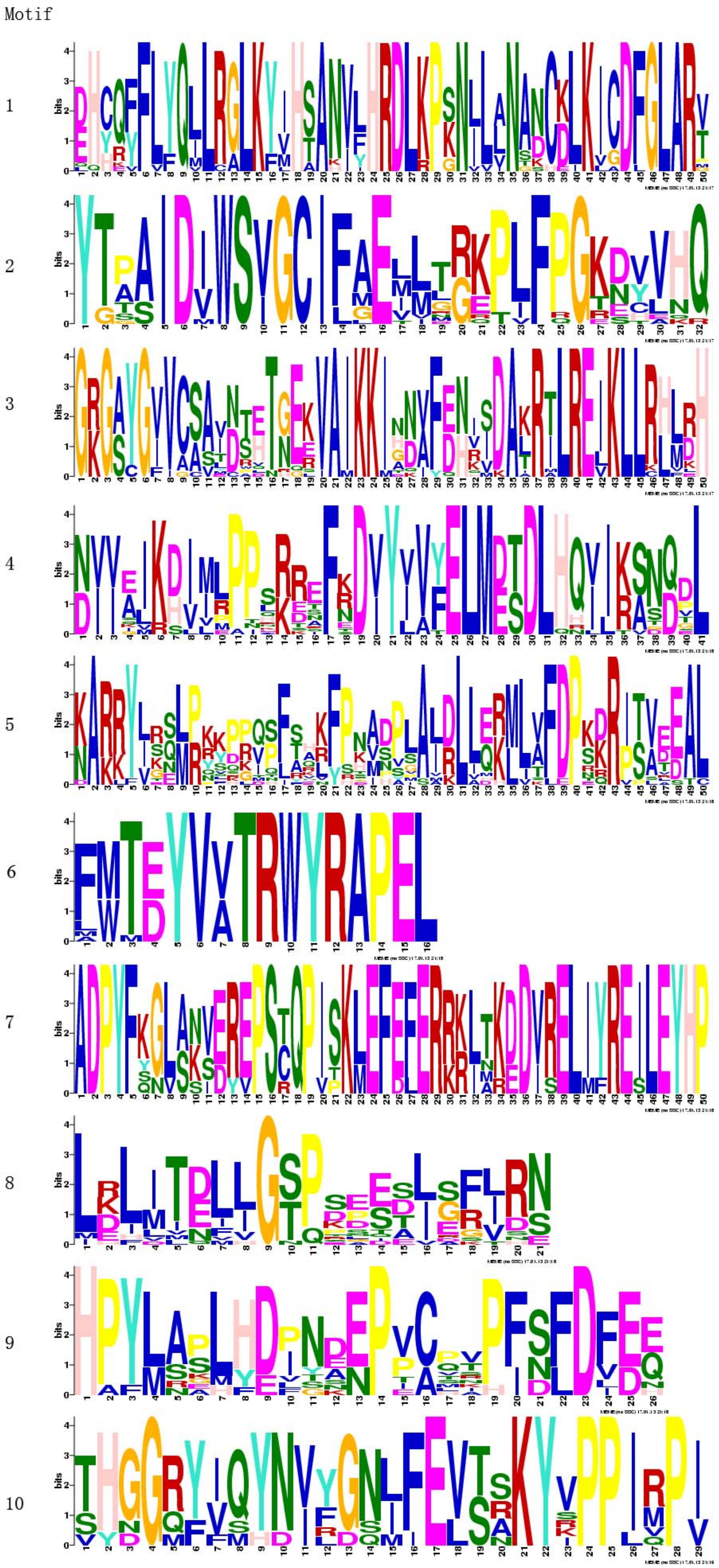

b

```
BnaMPK8     1 MGGGGNLVDGVRRWLFQRPSSSSSSNNNNPQEPIKKSDTFSNPDHQSELVITEDLDFSGL
BnaMPK9     1 ------------------------------------------------------------
BnaMPK17    1 ------------------------------------------------------------
BnaMPK16    1 ------------------------------------------------------------
BnaMPK19    1 ------------------------------------------------------------
BnaMPK20    1 ------------------------------------------------------------
BnaMPK1     1 ------------------------------------------------------MATPVD
BnaMPK2     1 ------------------------------------------------------MATPVD
BnaMPK3     1 ---------------------------------------------------MNNAGGQYP
BnaMPK6     1 -------------------------------------MDGGTGQPAADTEMSEVTGGAAP
BnaMPK4     1 ------------------------------------------------------MSAENC
BnaMPK5     1 ------------------------------------------------------MSSETE
consensus   1

                               Motif 10                 Motif 3
BnaMPK8    61 KLIKVPKRHHLPMDPQKKGVQEKDFFTEYGEAN--RYQVQEVVGKGSYGVVASALDTHTG
BnaMPK9     1 ------------MDPHKKVTLETEFFTEYGEAS--RYQIQEVIGKGSYGVVASAIDTHTG
BnaMPK17    1 -------------------MLEKEFFTEYGEAS--QYQIQEVVGKGSYGVVASAECPHTG
BnaMPK16    1 ----------MQPDQRKKSSVELDFFTEYGEGT--RYRIEEVIGKGSYGVVCSAYDTHTG
BnaMPK19    1 ----------MQQGQVKKNMKEVDFFTEYGDAN--RYQILEVIGKGSYGVVCAAIDTHTG
BnaMPK20    1 ---------MQQDPQRKKNNLEMEFFSDYGDAS--RFKIQEVIGKGSYGVVCSAIDTLTG
BnaMPK1     7 P--------PNGVRNQGKHYFT--MWQNLFEIDTKYMP-IKPIGRGAYGVVCSSVNTDNN
BnaMPK2     7 P--------PNGVRNQGKHYFS--MWQTLFEIDTKYVP-IKPIGRGAYGVVCSSVNRETN
BnaMPK3    10 --------DFPAVQTHGGQFISYDIFGSLFEVTSKYRPPIVPIGRGAYGIVCSVLDSETN
BnaMPK6    24 SQPMPGIENIPATLSHGGMFIQYNIFGNIFEVTAKYSPPIMPIGKGAYGIVCSAMNSETN
BnaMPK4     7 FGGGGGDQSTKGLATHGGQYVQYNVYGNIFEVTRKYVPPLRPIGRGAYGIVCAATNSETG
BnaMPK5     7 S---ATENNIKGVLVHGGRYFQHNVYGNMFEVSNKYVPPIRPIGRGAYGFVCAAVDSETH
consensus  61            ..  ... ... ...... . . ....... .*.* **.*.........

                     Motif 3                            Motif 4
BnaMPK8   119 ERVAIKKINDVFEHVSDATRILREIKLLRLLRHPDVVEIKHIMLPPSRREFRDIYVVFEL
BnaMPK9    47 EKVAIKKINDVFEHVSDATRILREIKLLRLLRHPDIVEIKHVMLPPSRREFRDIYVVFEL
BnaMPK17   40 GKVAIKKMTNVFEHVSDAIRILREIKLLRLLKHPDIVEIKHIMLPPCRKEFKDIYVVFEL
BnaMPK16   49 EKVAIKKINDIFEHVSDATRILREIKLLRLLRHPDIVEIKHILLPPSRREFRDIYVVFEL
BnaMPK19   49 EKVAIKKINDVFEHISDALRILREVKLLRLLRHPDIVEIKSIMLPPSKREFKDIYVVFEL
BnaMPK20   50 EKVAIKKIHDIFEHISDAARILREIKLLRLLRHPDIVEIKHIMLPPSRREFKDIYVVFEL
BnaMPK1    56 EKVAIKKIHNVYENRIDALRTLRELKLLRHLRHENVIALKDVMMPIHKRSFKDVYLVYEL
BnaMPK2    56 ERVAIKKIHNVFQNRIDALRTLRELKLLRHLRHDNVIALKDVMMANHKRTFKDVYLVYEL
BnaMPK3    62 ELVAMKKIANAFDNHMDAKRTLREIKLLRHLDHENIIAIRDVVPPPLRRQFSDVYIATEL
BnaMPK6    84 ESVAIKKIANAFDNKIDAKRTLREIKLLRHMDHENIVAIRDIIPPPLRTAFNDVYIAYEL
BnaMPK4    67 EEVAIKKIGNAFDNIIDAKRTLREIKLLKHMDHENVIAVKDIIRPPLRENFNDVYIVYEL
BnaMPK5    64 EEIAIKKIGKAFDNKVDAKRTLREIKLLRHLEHENVVIIKDIIRPPKKETFTDVYIVYEL
consensus 121 ...*.**. .... ..** * ***.***. ..*. ........... ...*.*.*...**

                 Motif 4                       Motif 1
BnaMPK8   179 MESDLHQVIKANDDLTPDHYQFFLYQLLRGLKYVHAANVFHRDLKPKNILANADCKLKIC
BnaMPK9   107 MESDLHQVIKANDDLTPEHYQFFLYQLLRGLKFIHTANVFHRDLKPKNILANSDCKLKIC
BnaMPK17  100 MESDLHHVLKVNDDLTPQHHQFFLYQLLRGLKFMHSAHVFHRDLKPKNILANADCKIKIC
BnaMPK16  109 MESDLHQVIKANDDLTPEHYQFFLYQLLRGLKYIHTANVFHRDLKPKNILANADCKLKIC
BnaMPK19  109 MESDLHQVIKANDDLTKEHHQFFLYQMLRALKYMHTANVYHRDLKPKNILANANCKLKVC
BnaMPK20  110 MESDLHQVIKANDDLTKEHYQFFLYQLLRALKYIHTANVYHRDLKPKNILANANCKLKIC
BnaMPK1   116 MDTDLHQIIKSSQVLSNDHCQYFLFQLLRGLKYIHSANILHRDLKPGNLLINANCDLKIC
BnaMPK2   116 MDTDLHQIIKSSQVLSNDHCQYFLFQLLRGLKYIHSANILHRDLKPGNLLVNANCDLKIC
BnaMPK3   122 MDTDLHQIIRSNQGLSEEHCQYFLYQLLRGLKYIHSAKVIHRDLKPSNLLLNANCDLKIC
BnaMPK6   144 MDTDLHQIIRSNQGLSEEHCQYFLYQILRGLKYIHSANVLHRDLKPSNLLLNANCDLKIC
BnaMPK4   127 MDTDLHQIIRSNQPLTDDHCRFFLYQLLRGLKYVHSANVLHRDLKPSNLLLNANCDLKLG
BnaMPK5   124 MDTDLHQIIRSDQPLTDDHCQYFLYQILRGLKYIHSANVLHRDLKPSNLLLNANCDLKIA
consensus 181 *..***...... .*. .*...**.*.**.**..*.*.. ******.*.*.*..* .*..
```

```
                       Motif 1            Motif 6                  Motif 2
BnaMPK8    239 DFGLARVSFNDAPTAIFWTDYVATRWYRAPELCGSFFSKYTPAIDIWSVGCIFAEMILGK
BnaMPK9    167 DFGLARVSFNDAPSAIFWTDYVATRWYRAPELCGSFFSKYTPAIDIWSIGCIFAEMLTGK
BnaMPK17   160 DLGLARVSFTDSPSAVFWTDYVATRWYRAPELCGSFYSNYTPAIDMWSVGCIFAEMLTGK
BnaMPK16   169 DFGLARVAFNDTPTAIFWTDYVATRWYRAPELCGSFFSKYTPAIDIWSIGCIFAELLTGK
BnaMPK19   169 DFGLARVSFNDTPTTVFWTDYVATRWYRAPELCGSFCSKYTPAIDIWSIGCIFAEVLLGK
BnaMPK20   170 DFGLARVAFNDTPTTIFWTDYVATRWYRAPELCGSFYSKYTPAIDIWSIGCIFAEVLMGK
BnaMPK1    176 DFGLARTSN---TKGQLMTEYVVTRWYRAPELL-LCCDNYGTSIDVWSVGCIFAELLGRK
BnaMPK2    176 DFGLARMSN---TKGQFMTEYVVTRWYRAPELL-LCCDNYGTSIDVWSVGCIFAELLGRK
BnaMPK3    182 DFGLARPT----SENEFMTEYVVTRWYRAPELL-LNSSDYTAAIDVWSVGCIFMELMNRK
BnaMPK6    204 DFGLARVN----SESDFMTEYVVTRWYRAPELL-LNSSDYTAAIDVWSVGCIFMELMDRK
BnaMPK4    187 DFGLARTK----SETDFMTEYVVTRWYRAPELL-LNCSEYTAAIDIWSVGCILGETMTRE
BnaMPK5    184 DFGLARTT----SETDLMTEYVVTRWYRAPELL-LNSSEYTSAIDVWSVGCIFAEIMTRE
consensus  241 *.****... . ..... *.** ********* . . . *...**.**.***..*..  .

                 Motif 2            Motif 8                 Motif 5
BnaMPK8    299 PLFPGEDVVHQLDHMTDFHGNPPPESISRIRNEKARRYLSSMRKQHPDSFYHKFHKADPL
BnaMPK9    227 PLFPGKNVVHQLDIMTDLLGTPSPEAISRIRNEKARRYLGNMRRKPPVPFTHKFPHVDPL
BnaMPK17   220 PLFPGKNVVHQLELVTDLLGTPSPITLSRIRNEKARKYLSNMRRKDPVPFTHKFPNIDPV
BnaMPK16   229 PLFPGKNVVHQLDLMTDMLGTPSAEAIGRVRNEKARRYLSSMRKKKPIPFSHKFPHADPL
BnaMPK19   229 PLFPGKSVVHQLELITDLLGTPKSETIAGVRNEKARKYLGEMRKKSLVPFTQKFPNADPS
BnaMPK20   230 PLFPGKNVVHQLDLMTDLLGTPSLDTISRVRNEKARRYLTSMRKKPPIPFTQKFPNADPL
BnaMPK1    232 PIFQGTECLNQLKLIVNILGSQRDEDLEFIDNPKAKRYIRSLPYSPGMSLSRLYPGAHVL
BnaMPK2    232 PIFPGTECLNQIKLIINILGSQKEEDLEFIDNPKAKRYIESLPYSPGISFSRLYPNANVL
BnaMPK3    237 PLFPGKDHVHQMRLLTELLGTPTESDLGFTHNEDAKRYIRQLPNFPRQPLAKLFSHVNPL
BnaMPK6    259 PLFPGRDHVHQLRLLMELIGTPSEQEVEFLN-ENAKRYIRQLPPYPRQSITDKFPNVHPL
BnaMPK4    242 PLFPGKDYVHQLRLITELIGSPDDSSLGFLRSDNARRYVKQLPQYPRQNFAARFPNMSAG
BnaMPK5    239 PLFPGKDYVHQLKLITELIGSPDGTSLEFLRSENARKYVKELPKLPRQSFAARFPSMSSI
consensus  301 *.*.*.....*. ......*... ... ......*..*.  . . . .............

                                         Motif 9
                      Motif 5                  Motif 7
BnaMPK8    359 ALRLLERLITFDPKDRVSADDALADPYFSGLSNSERVPSTQPISKLEFDLERKKLNKDDV
BnaMPK9    287 ALRLLHRLLAFDPKDRPTAEEALADPYFYGLANVDREPSTQPIPKLEFEFERRKIMKEDV
BnaMPK17   280 ALKLLQHLVAFDPKDRPSAEEALADPYFQGLANVDYEPSRQPISKLEFEFERRKLTRDDV
BnaMPK16   289 ALRLLEKMLSFEPKDRPTSEEALADPYFKNLAKVEREPSCQPVTKLEFEFERRRITKEDV
BnaMPK19   289 ALRLLQRLLAFDPKDRPTATEALADPYFKGLAKVEREPSCQPISKMEFEFERRRLAKDDI
BnaMPK20   290 SLKLLERLLAFDPKDRP-PLRSTCDPYFKDCESGEG-ASCQPIR-DEFEFERRKVTKEDI
BnaMPK1    292 AIDLLQKMLVFDPSKRISVTEALQHPYMAPLYDPNANPPAQVPIDLDVD---EELGEEMI
BnaMPK2    292 AIDLLQKMLVLDPSKRISVVEALQHPYMAPLYDPNANPPAQVPIDLDVDED-EDLGAEMI
BnaMPK3    297 AIDLVDRMLTFDPNKRITVEEALNHPYLAKLHDPNDEPICLKPFSFDFEQ--QPLDEEQI
BnaMPK6    318 AIDLIEKMLTFDPRRRITVLDALAHPYLNSLHDISDEPECTIPFNFDFEQ--HALSEEQM
BnaMPK4    302 AADLLEKMLVFDPSRRITVDEALCHPYLAPLHDINEEPVCVRPFNFDFEQ--PSLTEENI
BnaMPK5    299 AIDLLEKMLVFDPEKRITVEEALCHPFLSALHDLNDEPVCSNHFSFDFEN--PSSTEEEI
consensus  361 ...*..... ..*..*........ *..  . ... .......  .... ..........

                  Motif 7
BnaMPK8    419 SELIFREILEYHPQMMQEYKRGGDQVS--SMSPTGVDRFKRQFAHLEENQGKPGAGAGGG
BnaMPK9    347 RELIYREILEYHPRMLQEYLRGGEQTS--FMYPSGVDRFKRQFAHLEEHYGK------GE
BnaMPK17   340 RELMYREILEYHPRMLQEYLQGEENINSHFMYPSGVDQFKQEFARLEEHDDN----EEER
BnaMPK16   349 RELIYRESLEYHPKMLKEYLDGSEPTN--FMYPSAVEHFKKQFAYLEEHYKN-----GTS
BnaMPK19   349 RELIYREILEYHPQLLKDYMNGSEGSS--FLYPSAIGHLRKQFAYLEENSGK------GG
BnaMPK20   347 RELISREILEYHPQLLKDHMSGADKTN--FLYPSAVDQFRRQFAHLEENSGK------TG
BnaMPK1    349 REMMWNEMLHYHPQASPFELL---------------------------------------
BnaMPK2    351 RELMWEEMVHYHPETVTLSSILVY------------------------------------
BnaMPK3    355 KEMIYREAIALNPTYA--------------------------------------------
BnaMPK6    376 KELIYREALAFNPEYQPAIA----------------------------------------
BnaMPK4    360 KELIYRETVKFNPQ----------------------------------------------
BnaMPK5    357 KELVWLESVKFNPHPTN-------------------------------------------
consensus  421 .*....*.....* .. . . . .      . ....   .  .. ...
```

```
BnaMPK8    477 RSTAMHRHHASLPRERVPAQSGETAEESS-------------------------------
BnaMPK9    399 RSAPLQQRHASLPRERVPAPKEENRPAAT-------------------------------
BnaMPK17   396 NSPPIQRKYTSLPRERVCSSEDEGGDSVH-------------------------------
BnaMPK16   402 HNPPERQQHASLPRACVLYSDNNHAAAQQSSVEVTDGLSKCSIRDTERPRGADRRVPINV
BnaMPK19   401 PIIPPERKHASLPRSTVHPGVVTTSNAQPPESRRHVSFDPSRNVVPSTSTKPLGPPPRIP
BnaMPK20   399 PVAPLERKHASLPRSTVIHSTGVARGGQPKLMNSTNTLNPETTRNIPLNHHATLQAQQRN
BnaMPK1        ------------------------------------------------------------
BnaMPK2        ------------------------------------------------------------
BnaMPK3        ------------------------------------------------------------
BnaMPK6        ------------------------------------------------------------
BnaMPK4        ------------------------------------------------------------
BnaMPK5        ------------------------------------------------------------
consensus  481           ....  .

BnaMPK8    506 ------DVERR---------------------------------AAAAVASTLESEEAD-
BnaMPK9    428 ------LATTP---------------------------------ESPQSSQHEGSNYMNG
BnaMPK17   425 ------AQLPS---------------------------------SSVVFTPPQTPNTETG
BnaMPK16   462 PQTIQGAAVAR---------------------------------PGKVVGSVLRYNNCGA
BnaMPK19   461 SGRPGRAVESSSLTYENNRNLKESSSYDARTSYYRTTPQQNTVSPNCFFNPNTTMNQEKR
BnaMPK20   459 LSAAKPSAFMG--PVAPFDNGRSSRDAYDPRSFIRSTTTSLPFQQQSASTAAMGKQQERR
BnaMPK1        ------------------------------------------------------------
BnaMPK2        ------------------------------------------------------------
BnaMPK3        ------------------------------------------------------------
BnaMPK6        ------------------------------------------------------------
BnaMPK4        ------------------------------------------------------------
BnaMPK5        ------------------------------------------------------------
consensus  541

BnaMPK8    526 -NGGGYSARSLMKS------------SSISGSKCIGVQSKTDKEDTIAEEGDGESVAELT
BnaMPK9    449 MSQTGYSARSLLKS------------ASISASKCIGVKQRNQSE---HGESNSDATDALS
BnaMPK17   446 LSSKKTAQVDKAAA------------TPVKRSACLVR-------------SDSICASRCV
BnaMPK16   489 ATGVEAIEQQQRRM------------VRNPAGAAQYPKRTPQPCKSNRGDEDSEGSSRLK
BnaMPK19   521 LGTEAAASQSKPQF------------VPTQCNSAKPAELNPNPYVQQTQHKVGIDAKLLH
BnaMPK20   517 TTTTMESEKQARQISQYNIYAQPDVAINIDNNPFIMARTGMHKAENMSDHRIIIDTNLLQ
BnaMPK1        ------------------------------------------------------------
BnaMPK2        ------------------------------------------------------------
BnaMPK3        ------------------------------------------------------------
BnaMPK6        ------------------------------------------------------------
BnaMPK4        ------------------------------------------------------------
BnaMPK5        ------------------------------------------------------------
consensus  601

BnaMPK8    573 DRV-ASLRNS-------------------------
BnaMPK9    494 QKA-AALHT--------------------------
BnaMPK17   481 GVS-SAVS---------------------------
BnaMPK16   537 QNP-QYIPQKVGGAQDTATSRWY------------
BnaMPK19   569 AQS-QYGPAGAAAVAVAVAAHRSVGTVGYGMS---
BnaMPK20   577 ATAGIGVAAAAAAAAPGGSAHRKVGGVRYGMSKMY
BnaMPK1        -----------------------------------
BnaMPK2        -----------------------------------
BnaMPK3        -----------------------------------
BnaMPK6        -----------------------------------
BnaMPK4        -----------------------------------
BnaMPK5        -----------------------------------
consensus  661
```

Figure S5. A detailed motif analysis and multiple alignment of canola MPKs.
a, The analysis is from MEME 4.9.0 (Release date: Wed Oct 3 11:07:26 EST 2012).
Stack heights represent conservation at a position, and symbol heights within a stack represent the relative frequency of each residue.
b, Multiple alignment of BnaMPKs with 10 motifs shown as overbars.
